# Sequencing HIV-neutralizing antibody exons and introns reveals detailed aspects of lineage maturation

**DOI:** 10.1038/s41467-018-06424-6

**Published:** 2018-10-08

**Authors:** Erik L. Johnson, Nicole A. Doria-Rose, Jason Gorman, Jinal N. Bhiman, Chaim A. Schramm, Ashley Q. Vu, William H. Law, Baoshan Zhang, Valerie Bekker, Salim S. Abdool Karim, Gregory C. Ippolito, Lynn Morris, Penny L. Moore, Peter D. Kwong, John R. Mascola, George Georgiou

**Affiliations:** 1 Department of Chemical Engineering, The University of Texas at Austin, Austin, TX 78712 USA; 2 Vaccine Research Center, National Institute of Allergy and Infectious Diseases, National Institutes of Health, Bethesda, MD 20892 USA; 3 Center for HIV and STIs, National Institute for Communicable Diseases of the National Health Laboratory Service (NHLS), Johannesburg, 2131 South Africa; 4 Faculty of Health Sciences, University of the Witwatersrand, Johannesburg, 2050 South Africa; 5 Department of Molecular Biosciences, The University of Texas at Austin, Austin, TX 78712 USA; 6 Centre for the AIDS Programme of Research in South Africa (CAPRISA), University of KwaZulu-Natal, Congella, 4013 South Africa; 7 Department of Epidemiology, Columbia University, New York, NY 10032 USA; 8 Department of Biomedical Engineering, The University of Texas at Austin, Austin, TX 78712 USA; 9 Present Address: The University of Texas Southwestern Medical Center, Dallas, TX 75390 USA; 10 Present Address: Geisel School of Medicine, Dartmouth College, Hanover, NH 03755 USA

## Abstract

The developmental pathways of broadly neutralizing antibodies (bNAbs) against HIV are of great importance for the design of immunogens that can elicit protective responses. Here we show the maturation features of the HIV-neutralizing anti-V1V2 VRC26 lineage by simultaneously sequencing the exon together with the downstream intron of VRC26 members. Using the mutational landscapes of both segments and the selection-free nature of the intron region, we identify multiple events of amino acid mutational convergence in the complementarity-determining region 3 (CDR3) of VRC26 members, and determine potential intermediates with diverse CDR3s to a late stage bNAb from 2 years prior to its isolation. Moreover, we functionally characterize the earliest neutralizing intermediates with critical CDR3 mutations, with some emerging only 14 weeks after initial lineage detection and containing only ~6% V gene mutations. Our results thus underscore the utility of analyzing exons and introns simultaneously for studying antibody maturation and repertoire selection.

## Abstract (web summary)

Knowledge on how antibody responses have evolved is critical for the induction of protective immunity. Here the authors analyse, using high-throughput sequencing of both exon and intron regions, the mutation and lineage development of an HIV-neutralizing antibody to find an unexpected early emergence of broadly neutralizing species.

## Introduction

The elicitation of HIV-1-specific broadly neutralizing antibodies (bNAbs) represents a major challenge^1,2^. A detailed understanding of how bNAbs mature from naive precursors in HIV-1-infected individuals has the potential to aid in the design of vaccine immunogens^3,4^. Specifically, reconstructing the timeline of the emergence of molecular properties that confer neutralization breadth and identifying relevant intermediate antibodies bearing these traits may provide chronological templates for immunogen design^5,6^. These phylogenetic timelines are typically reconstructed from heavy chain exonic variable region (V_H_) sequences obtained by the next-generation sequencing of peripheral B cells^7–10^. However, the interpretation of antibody developmental pathways can be confounded by a number of issues including: (a) the presence of sequences with diverse heavy chain CDR3s (CDRH3s)^11^, (b) inferring the correct unmutated common ancestor (UCA)^5,10^, (c) multiple mutations at the same site or convergent mutations^12^, (e) under-sampling^13^, (f) sequencing and PCR error^14^, (g) computational phylogenetic limitations^15^ and (h) the inappropriate use of binary trees for antibody phylogeny^16^.

To remove as much uncertainty as possible in interpreting lineage evolution, we developed an approach that capitalizes not only on the sequence of the antibody exon but also on the downstream intron between the J and C genes (J/C intron). Extensive studies have established that somatic hypermutation (SHM) continues for more than 1 kb downstream of the variable region exon into the J/C intron. Mutations occur in the exon and intron at the same rate of 1 mutation per ~10^3^ bp per cell division^17–20^. Importantly, unlike exonic mutations which are subjected to selection for expression, folding, and antigen binding, mutations in the intron are not subject to selective pressure^17,21–25^.

Here, we sought to utilize this methodology to identify previously unrecognized developmental characteristics of the HIV-specific anti-V1V2 VRC26 bNAb lineage which was isolated from donor CAP256 and sampled for more than 4 years^7,26,27^. First, we aimed to discover and better understand the maturation timeline of early functional intermediates preceding the emergence of long CDRH3^−^stabilizing mutations and the development of the broadest known VRC26 bNAbs. These intermediates might be used for immunogen design but also to better delineate when neutralization breadth began to arise and the minimum V_H_ gene mutation levels for achieving this breadth in this critical lineage subsection. Second, we investigated whether developmental complexities such as intraclonal mutational convergence were occurring since there is no standard means for characterizing and confirming the presence of these phenomena using an exon alone, particularly when under-sampling is likely. Third, we wanted to better understand the phylogenetic relationship between VRC26 antibodies with highly diverse CDRH3s, particularly for confirming additional intermediates. This has been a significant challenge in the case of other HIV bNAb lineages^10,28^.

Here the use of both exon and intron phylogenies provides a distinct mechanism for studying these detailed maturation characteristics of the VRC26 lineage. The sequencing and informatics analysis of the mutational landscape within these two genetic segments enables the identification of CDRH3 amino acid mutations that emerge independently in multiple VRC26 sublineages and provides a better understanding of the phylogenetic relationship among bNAbs with highly divergent CDRH3s and their intermediates. Importantly, the study of these two phylogenies also resulted in the selection of early intermediate antibodies that display increasing affinity for an autologous viral strain and increasing heterologous breadth within 14 weeks of the initial detection of the VRC26 lineage with only ~6% mutated V_H_ gene. This approach provides a high resolution of bNAb evolutionary trajectories that may be applied to HIV-1 immunogen design and beyond.

## Results

### Exonic and intronic mutations accumulate in the VRC26 lineage

**Fig. 1:**
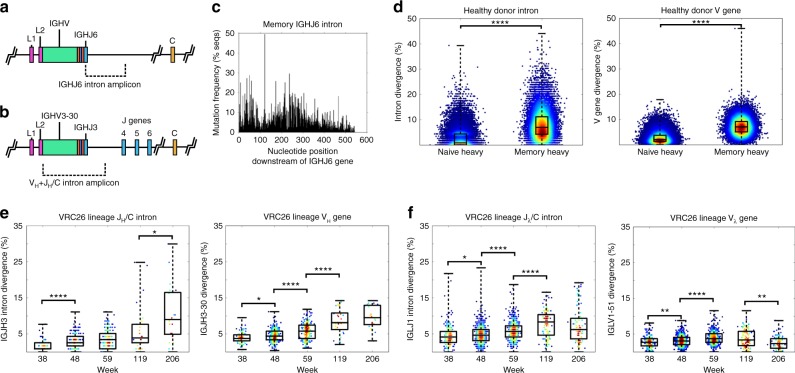
Exonic and intronic mutations accumulate in healthy donor and VRC26 B cells. **a** Diagram of a human heavy chain locus with a rearranged IGHJ6 gene and the IGHJ6 intron amplicon source. **b** A human heavy chain locus with a rearranged IGHJ3 gene with an example V_H_ + J_H_/C intron amplicon source indicated. All V_H_ + J_H_/C VRC26 heavy chain amplicons are of the IGHJ3 rearrangement. **c** The intronic mutation distribution across a ~550 bp segment directly downstream of the J gene of IGHJ6-rearranged antibodies from healthy donor mBCs (number of sequences (*n*) = 4796). **d** Germline divergence of V_H_ + J_H_/C sequences from naive B cells and mBCs from a healthy donor. Intron divergence is shown (left) as well as the corresponding V gene divergence (right) (Naive Heavy: *n* = 47,372, Memory Heavy: *n* = 19,704). **e** Intron divergence (left) and V gene divergence (right) for VRC26 heavy chains at all longitudinal time points (week 38: *n* = 78, week 48: *n* = 240, week 59: *n* = 193, week 119: *n* = 30, week 206: *n* = 20). **f** Intron divergence (left) and V gene divergence (right) for VRC26 light chains at vall longitudinal time points (week 38: *n* = 184, week 48: *n* = 807, week 59: *n* = 570, week 119: *n* = 110, week 206: *n* = 85). Points are colored by point density using the Python Gaussian kernel density estimator function for visualization. Two-tailed Mann–Whitney *U* test *p*-values are shown (****p-value ≤ 0.0001, ***p-value ≤ 0.001, **p-value ≤ 0.01, *p-value ≤ 0.05). Center lines on boxplots represent the median, while the box limits represent the upper and lower quartiles and whiskers show the maximum and minimum values

To assess the extent and frequency of intronic mutations in healthy individuals, we sequenced an ~550 bp segment of the heavy chain J/C intron (J_H_/C intron) directly downstream from heavy chain sequences with rearranged immunoglobulin heavy chain J gene 6 (IGHJ6) genes starting from ~100,000 FACS-sorted memory B cells (mBCs; CD3^-^CD20^+^CD19^+^IgD^−^CD27^+^) (Fig. 1a). Some sites exhibited a mutation frequency of >10% up to about 450 bp downstream into the intron (Fig. 1c). The genomic DNA (gDNA) of FACS-sorted naive B cells (259,000 cells; CD3^−^CD20^+^CD19^+^IgD^+^CD27^−^) and mBCs (219,000 cells) from a healthy donor was then used to amplify all V_H_ antibody sequences extending from the framework region 1 (FR1) to about 140 bp into the J_H_/C intron (Fig. 1b, Supplementary Tables 1 and 2) (denoted from here on as V_H_ + J_H_/C amplicons). As expected, the mutation frequency in both the V_H_ gene and J_H_/C intron segments were substantially greater in the mBC repertoire relative to the naive repertoire, with intron segments possessing overall higher levels of mutations (Fig. 1d, Supplementary Fig. 1).

We applied this amplification scheme to generate gDNA-derived V_H_ + J_H_/C and analogous lambda light chain V_λ_ + J_λ_/C amplicons (extending from the FR1 to ~230 bp into the J_λ_/C intron) for antibodies that are putative members of the VRC26 HIV-1-specific bNAb lineage from donor CAP256. Genomic DNA from weeks 38, 48, 59, 119 and 206 after primary infection (Supplementary Table 1) was used as template. The VRC26 lineage was likely elicited by superinfecting viral variants at week 34 after primary infection^7,26,29^. Antibody sequences using the VRC26 germline immunoglobulin heavy chain V gene 3–30 (IGHV3–30) and germline immunoglobulin heavy chain J gene 3 (IGHJ3) and having ≥85% CDRH3 nucleotide identity to earlier published VRC26 antibodies^7,26^ were assumed to be members of the lineage (Supplementary Table 3). Light chain lineage members were required to use the VRC26 germline immunoglobulin lambda light chain V gene 1–51 (IGLV1–51) and immunoglobulin lambda light chain J gene 1 (IGLJ1) and have ≥92% CDRL3 identity to previously published members^7,26^ (Supplementary Table 4). V gene and intron mutation loads were plotted for the lineage sequences isolated at each time point. As expected, sequences bearing higher intronic divergence increased in number over time after infection. An increase in intronic mutational divergence (Mann–Whitney *U* test) was most apparent during the first few time points for both heavy and light chain sequences (Fig. 1e, f). While this finding indicates that diversification continued over time, negative selection was previously shown to increase as time progressed to possibly control for deleterious mutations due to this diversification^30^.

### CDRH3 mutational convergence in two unique sublineages

**Fig. 2:**
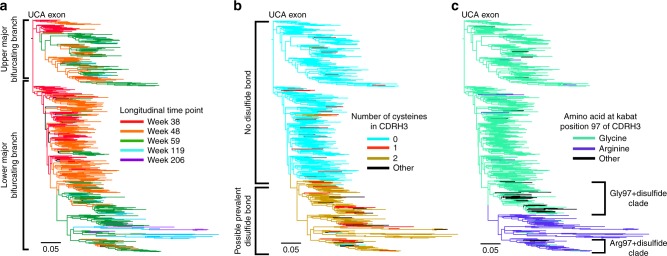
Critical CDRH3-stabilizing mutations of the VRC26 lineage. **a** The V_H_ exon ML tree with sequences highlighted by the longitudinal time point at which they were isolated. **b** The V_H_ exon ML tree showing the number of cysteines in the CDRH3. **c** Same as in **b** but colored with respect to the amino acid at CDRH3 Kabat position 97

A maximum-likelihood (ML)^15^ phylogenetic tree generated using the V_H_ exon segment of all putative lineage members and rooted on the previously inferred UCA^7^ contains two signature upper and lower major bifurcating branches^7,26^ (Fig. 2a). 31/33 VRC26 bNAbs previously isolated by single-cell sequencing, including all of the most potent and broad (46–63% heterologous neutralization breadth), fall within the lower major bifurcating branch^7^. Molecular traits essential for this broad neutralization and present in 25/31 of these lower branch bNAbs include a stabilizing disulfide bond in the CDRH3 (Cys100a and Cys100q mutations; Kabat numbering) and an Arg97 that has been proposed to further stabilize the CDRH3^7,26^. When the V_H_ ML tree is highlighted for the presence of the CDRH3 cysteines that give rise to the disulfide bond (Fig. 2b) or for the Arg97 mutation (Fig. 2c), two distinct sublineages in the lower major bifurcating branch become evident. One of these sublineages develops a disulfide bond and the Arg97 mutation (Arg97 + Disulfide sublineage), while members of the other sublineage gain a disulfide bond but instead generally retain the initial Gly97 of the UCA (Gly97 + disulfide sublineage). Additional mutations outside of the CDRH3 also serve to differentiate between these two sublineages (Supplementary Fig. 2).

**Fig. 3:**
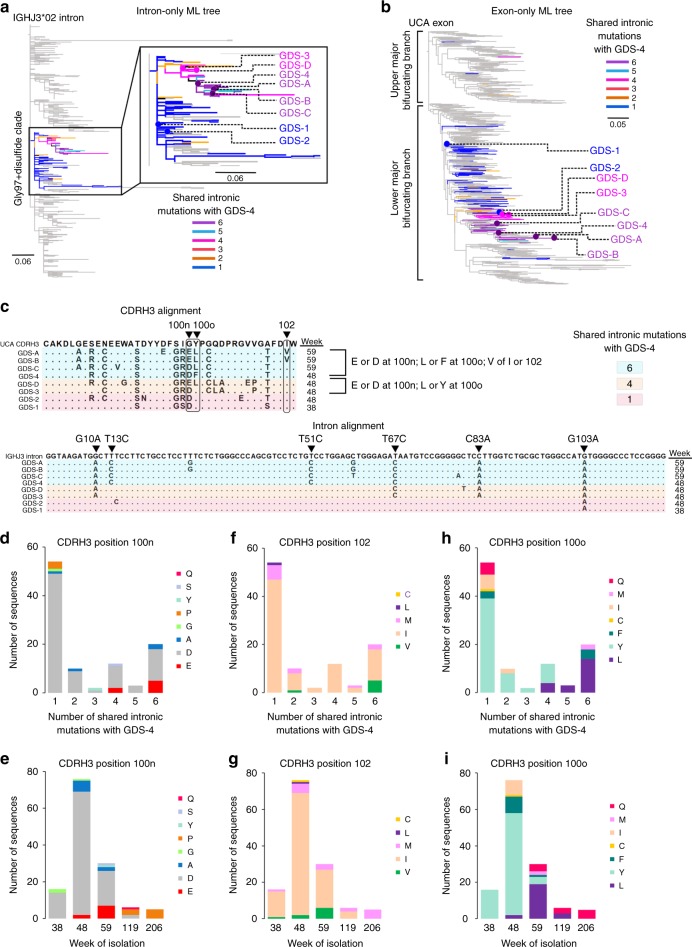
Development of convergent mutations within the Gly97 + disulfide sublineage. **a** Intron-only ML tree for all VRC26 lineage members. The Gly97 + disulfide clade is highlighted by number of shared intronic mutations with GDS-4. Sequences labeled GDS-(1–4) were selected for expression. **b** The exon-only ML tree for all VRC26 members is shown with the location of the highlighted sequences in **a** indicated. **c** CDRH3 and intron alignment for the representative sequences marked in **a** and **b**. Three CDRH3 sites that eventually develop convergent mutations are circled and longitudinal time points of isolation are also indicated. GDS-4 intronic mutations are labeled and sequences are colored by the number of these mutations that they possess. **d** Number of Gly97 + disulfide clade sequences that share intronic mutations with GDS-4 and their amino acid composition at CDRH3 Kabat position 100n. **e** Same as in **d** except shown by longitudinal time point of isolation. **f** Same as **d** except shown for CDRH3 position 102. **g** Same as in **e** except for CDRH3 position 102. **h** Same as in **d** except for CDRH3 position 100o. **i** Same as in **e** except for CDRH3 position 100o

**Fig. 4:**
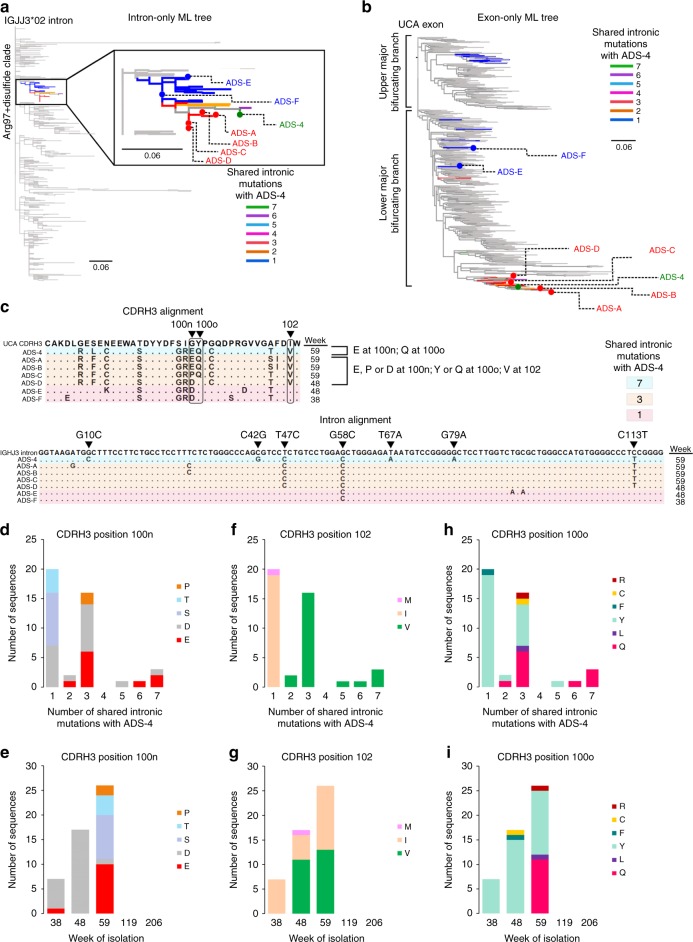
Development of convergent mutations within the Arg97 + disulfide sublineage. **a** Intron-only ML tree for all VRC26 lineage members. The Arg97 + disulfide clade is highlighted by number of shared intronic mutations with ADS-4. **b** The exon-only ML tree for all VRC26 members is shown with the location of the highlighted sequences in **a** indicated. **c** CDRH3 and intron alignment for the representative sequences marked in **a** and **b**. Three CDRH3 sites that eventually develop convergent mutations are circled and longitudinal time points of isolation are also indicated. ADS-4 intronic mutations are labeled and sequences are colored by the number of these mutations that they possess. **d** Number of Arg97 + disulfide clade sequences that share intronic mutations with ADS-4 and their amino acid composition at CDRH3 Kabat position 100n. **e** Same as in **d** except shown by longitudinal time point of isolation. **f** Same as **d** except shown for CDRH3 position 102. **g** Same as in **e** except for CDRH3 position 102. **h** Same as in **d** except for CDRH3 position 100o. **i** Same as in **e** except for CDRH3 position 100o

To investigate the phylogeny of these two sublineages in greater detail we also constructed a ML phylogenetic tree using the J_H_/C intron segment of all VRC26 lineage members and rooted it on the germline IGHJ3 allele 2 (IGHJ3*02) intron. To visualize the accumulation of intronic mutations and anchor our analysis, we selected an exceptionally high-quality sequence observed in more than one dataset with relatively mutated introns from each sublineage (GDS-4 for the Gly97 + Disulfide sublineage and ADS-4 for the Arg97 + Disulfide sublineage). We then examined the shared intronic mutations of all members of the corresponding clade (Figs. 3a, 4a) and marked the respective heavy chain sequences on the V_H_ exon tree (Figs. 3b, 4b).

The intronic mutations of the antibodies within these two sublineages define distinct maturation pathways (Figs. 3c, 4c), also reflected in the fact that the ADS-4 and GDS-4 exons share only three nucleotide substitutions in the V_H_ gene. Nonetheless, at least two CDRH3 amino acid mutations, Glu100n and Val102, appear to have emerged independently in both sublineages at later longitudinal time points (Figs. 3c, 4c). For example, since many sequences from the Gly97 + Disulfide sublineage (including GDS-4) that share at least 6 unique intronic mutations have either an Asp100n or Glu100n, one of these mutations likely preceded the other (Fig. 3c). Since the Glu100n appears later in the phylogeny in sequences with more shared intronic mutations with GDS-4 (Fig. 3d), and at later longitudinal time points (Fig. 3e), it likely appeared after the Asp100n. The Val102 also appears to have developed later in the Gly97 + disulfide sublineage (Figs. 3f, g). However, a similar pattern of later independent development is evident for the Glu100n and Val102 of the Arg97 + disulfide sublineage (Fig. 4c–g). Outside of the CDRH3, an Asp82a FR3 mutation also appears over time within each of these sublineages independently (Supplementary Fig. 3). These examples illustrate how the analysis of intronic mutations enables the identification of intraclonal convergence events within highly expanded antibody lineages.

### Discovery of early neutralizing intermediates

To better understand the development of these sublineages for neutralization capacity, we capitalized on our knowledge of both the J_H_/C intron and V_H_ exon phylogenies. We selected sequences from each sublineage for expression from multiple chronological stages of the selection-free intronic phylogeny that also had their relationship supported by the V_H_ exon tree. This is especially important for identifying sequences that might be very early members of each sublineage when they are difficult to differentiate from those of other sublineages. 31/33 of the previously isolated VRC26 bNAbs are known to be located on the lower major bifurcating branch and were found between week 119 and week 206, late in lineage development. In contrast, we used our analysis to select sequences to be the earliest (week 38–59) characterized intermediate antibodies on the lower major bifurcating branch. These antibodies would not only serve as functional intermediates but would provide more information about when and how neutralization properties developed in this critical section of the phylogeny that leads to the broadest and most potent bNAbs.

**Fig. 5:**
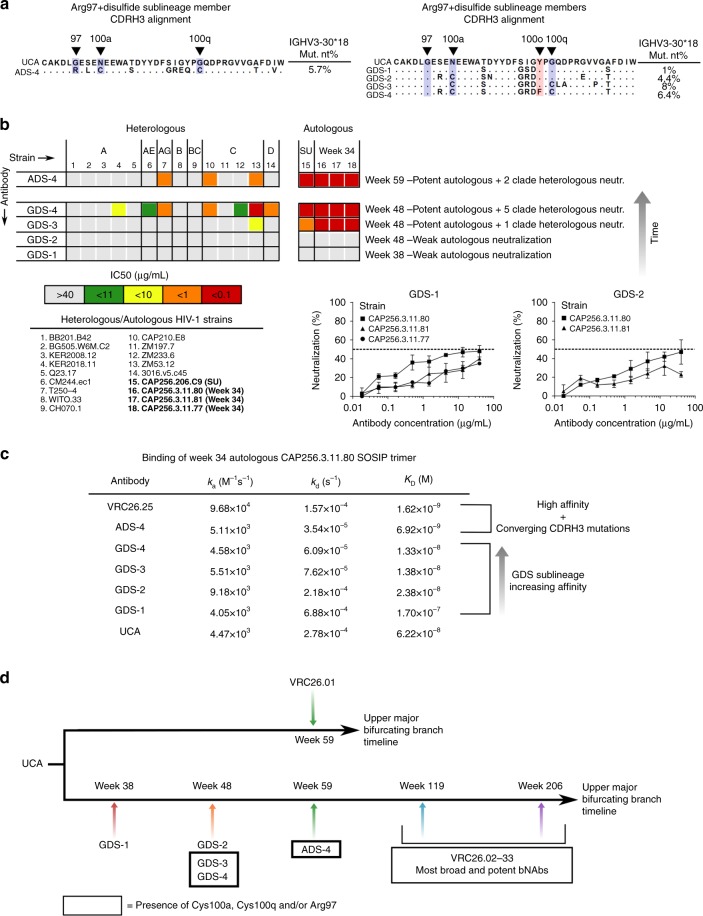
Discovery of early neutralizing intermediates with CDRH3-stabilizing mutations. **a** CDRH3 alignment for ADS-4 from the Arg97 + disulfide sublineage and GDS-(1–4) from the Gly97 + disulfide sublineage showing the critical CDRH3-stabilizing mutations. Percent nucleotide mutation for the V_H_ gene is shown. **b** Neutralization capacity for ADS-4 and GDS-(1–4). IC_50_ values (µg/mL) are shown and indicate a trend in increasing potency and breadth for the Gly97 + disfulide sublineage members. Strain names in bold are autologous and the superinfecting strain (SU) and the week 34 longitudinal time point strains are labeled. Neutralization curves for GDS-1 and GDS-2 antibodies showing weak neutralization of early appearing autologous strains from week 34 are shown. **c** Binding kinetics of week 34 autologous strain CAP256.3.11.80 to the synthesized antibodies, the UCA and VRC26.25. **d** Timeline of all tested antibodies from the VRC26 lineage shown for the two major bifurcating branches. All newly identified intermediates for the early stages of the lower major bifurcating branch are in bold. Boxed sequences contain the Cys100a, Cys100q and Arg97 CDRH3 mutations. Only the lower major bifurcating branch leads to the most potent and broad antibodies with stabilizing mutations. Note that VRC26.24, while isolated at week 193, is actually phylogenetically placed on the upper branch by exon analysis and lacks the three stabilizing mutations^26^

Selected antibodies were designated as either ADS (Arg97 + disulfide sublineage) or GDS (Gly97 + disulfide sublineage) and were recombinantly expressed to assess their potency and breadth (Fig. 5a). Light chains were chosen by matching similar heavy and light chain phylogenetic tree clades^31^ coupled with the balancing of intronic mutation loads between heavy and light chains under the assumption that both chains accumulate mutations at about the same rate (Methods; Supplementary Fig. 4). Recombinantly expressed antibodies were tested for neutralization of a 14-member heterologous strain subpanel. Importantly, ADS-4, isolated at week 59 from the Arg97 + disulfide sublineage, had both the Arg97 and the disulfide bond and neutralized 3/14 of the heterologous subpanel strains as well as three early week 34 autologous viruses with ~5.7% V gene nucleotide divergence (Fig. 5b).

Of the four antibodies that were expressed from the Gly97 + Disulfide sublineage, GDS-1 (week 38; having no CDRH3 cysteines) and GDS-2 (week 48; having one of the two CDRH3 cysteines), did not neutralize any of the heterologous HIV-1 strains tested but weakly neutralized week 34 autologous strains (Fig. 5b). In contrast, GDS-3 (week 48), which contains the CDRH3 disulfide bond, potently neutralized not only week 34 autologous strains but also one heterologous strain (1/14). GDS-4, detected at week 48 with the disulfide bond and with ~6.4% V gene nucleotide divergence from germline, potently neutralized the same autologous strains as GDS-3, and additionally neutralized 7/14 heterologous subpanel strains (Fig. 5b). GDS-4 has 7 CDRH3 mutations from the UCA other than the Cys100a and Cys100q, only one of which, Phe100o, was unique to GDS-4 among the synthesized GDS antibodies (Fig. 5a). Thus, GDS-4 represents the earliest arising antibody displaying appreciable heterologous neutralization breadth from the lower major bifurcating branch (Fig. 5d).

To investigate the affinity maturation of these early intermediates, their binding kinetics to an early week 34 autologous viral strain SOSIP Env trimer was assessed by SPR (Fig. 5c**)**. The affinity of the trimer for each antibody intermediate increases from GDS-1 to GDS-2 to GDS-3 to GDS-4, consistent with progressive maturation. In addition, ADS-4 and VRC26.25 (the broadest VRC26 bNAb), both of which have between 1 and 3 converging CDRH3 mutations, have the highest affinity.

### Sequences with diverse CDRH3s share intronic mutations

**Fig. 6:**
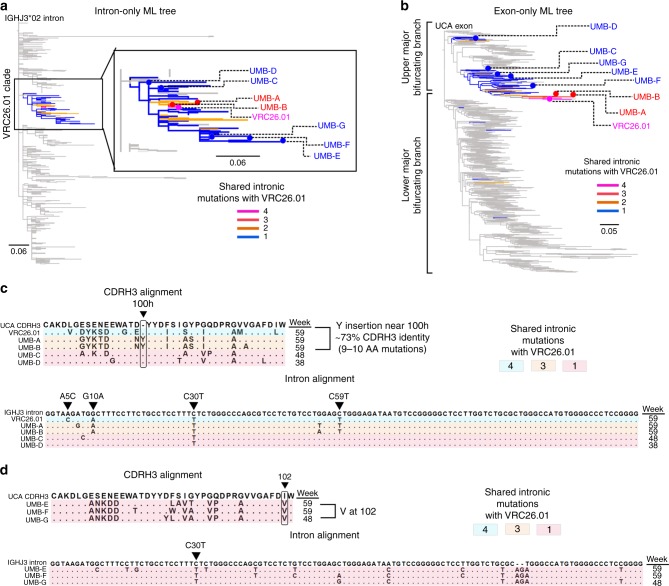
VRC26.01 sublineage members have diverse CDRH3s but share intronic mutations. **a** Intron-only ML tree for all VRC26 lineage members. The Arg97 + disulfide clade is highlighted by number of shared intronic mutations with ADS-4. Representative sequences are marked with UMB (Upper Major Bifurcating). **b** The exon-only ML tree for all VRC26 members is shown with the location of the highlighted sequences in **a** indicated. **c** CDRH3 and intron alignment for the representative sequences marked in **a** and **b**. Longitudinal time points of isolation are indicated. VRC26.01 intronic mutations are labeled and sequences are colored by the number of these mutations that they possess. UMB-A and UMB-B have a tyrosine position near position 100 h and have diverse CDRH3s from VRC26.01. **d** CDRH3 and intron alignment of sequences that diverge from VRC26.01 but share one intronic mutation. Longitudinal time points are shown and the CDRH3 site that contains convergent mutations is circled

The upper major bifurcating branch of the V_H_ exon tree contains a high quality sequence bearing the exon (differing in only 2 of the first 3 nucleotides of the FR1) of a previously-identified early NAb, VRC26.01, that was isolated at week 59 and neutralized 20% of the viruses tested (9/46)^7,26^. To investigate the VRC26.01 sublineage as we had with the Gly97 + disulfide and Arg97 + disulfide sublineages, we highlighted members of the VRC26.01 intronic clade based on the number of shared mutations with VRC26.01 (Fig. 6a; position in the VH exon tree in Fig. 6b).

Importantly, two antibodies from this sublineage contain a tyrosine insertion in the CDRH3 near Kabat position 100 h (Tyr100h), which is not present in VRC26.01. The CDRH3s of these Tyr100h antibodies are highly diverse and differ by up to 10 amino acids from VRC26.01 (~73% identity) (Fig. 6c). However, while the CDRH3s are divergent, these sequences share three intronic mutations and up to 14 non-CDRH3 V gene mutations with VRC26.01. The congruence of the intron and exon mutational landscapes indicate that despite having highly diverse CDRH3s, which notably included an amino acid insertion (i.e. Tyr100h), these antibodies likely diverged from the VRC26.01 pathway before the final A5C intronic mutation appeared. These sequences provide an important example of how intronic mutations can help further assess the phylogenetic relationship of members of a lineage with highly diverse CDRH3s and where they diverged.

Additional sequences from the same intronic clade that bear the same founder intronic C30T mutation but do not share any other intronic mutations with VRC26.01, may have branched off even earlier than the Tyr100h-containing antibodies. These early-diverging antibodies also eventually developed the Val102 CDRH3 mutation that appeared in both the Gly97 + disulfide and Arg97 + disulfide sublineages (Fig. 6d). The presence of antibody sequences having different intronic mutations from those of the Gly97 + disulfide and Arg97 + disulfide sublineages on an entirely different major bifurcating branch, reveals another intraclonal CDRH3 convergence event.

### Late-stage lineage maturation and intraclonal convergence

**Fig. 7:**
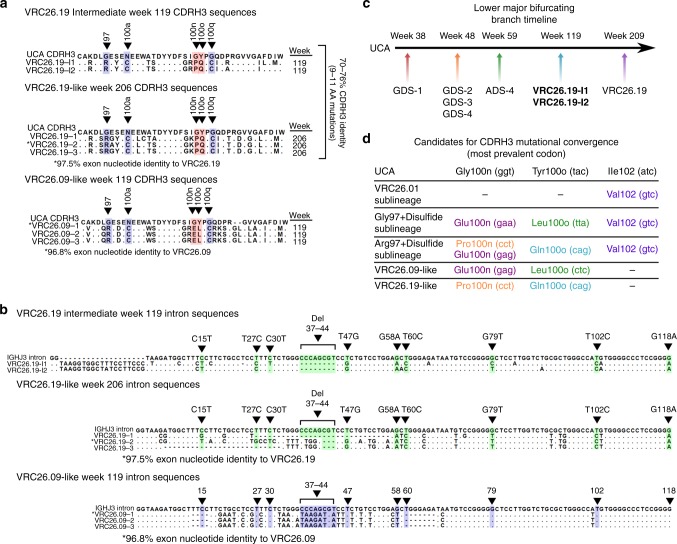
Late-stage bNAb intermediates and intraclonal convergence. **a** CDRH3 alignment of potential VRC26.19 intermediates (VRC26.19-I1, VRC26.19-I2), VRC26.19-like sequences (VRC26.19-(1–3)) and VRC26.09-like sequences (VRC26.09-(1–3)). VRC26.19-related sequences are highly diverse when compared between the two time points with about 70–76% CDRH3 amino acid identities between members of the two groups. CDRH3 sites containing residues that are candidates for intraclonal convergence throughout the lineage are marked in red. Sites with CDRH3-stabilizing mutations are marked in blue. **b** Alignment of the introns of the sequences shown in **a**. SHM events (point mutations or indels) that are shared between at least one member of each time point for VRC26.19-related sequences are marked with arrows and labeled according to germline positions. The same sites are marked for VRC26.09-like sequences for comparison. **c** Lower major bifurcating branch timeline with the potential intermediates for VRC26.19 indicated in bold. **d** List of sublineages and their candidate convergent CDRH3 mutations with the most prevalent codon shown

From the later week 206 time point after primary infection, we were able to find sequences within a 97.5% exonic nucleotide identity to the late-stage bNAb, VRC26.19, which was initially isolated by single-B cell cloning at week 206. This bNAb was previously shown to have a 46% neutralization breadth against 46 heterologous viruses^26^. Interestingly, while VRC26.19-like sequences were isolated 206 weeks after infection, the V_H_ exon and intron trees revealed VRC26.19-related antibodies in the week 119 blood sample (i.e. from nearly 2 years prior). While these week 119 antibody CDRH3s differ by as many as 11 residues from VRC26.19 (~70% amino acid CDRH3 identity) (Fig. 7a), there are 10 intronic SHM events (point mutations or indels) shared between at least one member of the VRC26.19-related week 119 and week 206 antibodies. Five of these mutations (T47G, G58A, T60C, T102C and G118A) occur in an aligned section of the introns devoid of indels and are shared among at least two sequences found at both time points (Fig. 7b). Some week 206 antibodies also share 23/36 V gene nucleotide mutations with week 119 antibodies. The congruence between the exon and intron phylogenies therefore help confirm potential heavy chain intermediates to the VRC26.19 bNAb, despite having diverse CDRH3s (Fig. 7c).

We also found sequences within a 96.8% exonic nucleotide identity to another bNAb, VRC26.09, previously isolated using single-B cell cloning at week 119. VRC26.09 was also shown to neutralize 46% of the heterologous strains tested (21/46)^7,26^. Although VRC26.19-like and VRC26.09-like sequences share ~7/36 exonic V gene mutations upstream of the CDRH3, their CDRH3’s are highly diverse (only ~50% amino acid identity), indicating that they diverged early on during lineage development (Fig. 7a). The introns of VRC26.19-like and VRC26.09-like antibodies share very few mutations, providing an additional genetic source supporting early divergence (Fig. 7b).

The intronic mutations of VRC26.19-like and VRC26.09-like sequences (Fig. 7b) are not only distinct from each other but are also different from those of members of the Gly97 + disulfide (Fig. 3c) and the Arg97 + disulfide sublineages (Fig. 4c). However, the CDRH3s across these four sets of antibodies share amino acid mutations that likely represent additional intraclonal convergence events including: Gln100o (Figs. 4c–h, i, 7a), Leu100o (Figs. 3c–h, i, 7a), Pro100n (Figs. 4c–e, 7a) and Glu100n (Figs. 3c–e, 4c–e, 7a). Like the Glu100n and Val102 mutations, Gln100o, Leu100o, and Pro100n also become more prevalent along the phylogeny of the Gly97 + disulfide and Arg97 + disulfide sublineages and with later longitudinal time points. Some of these convergent mutations utilize the same or slightly different nucleotide codons (Fig. 7d). However, the Leu100o of the Gly97 + disulfide sublineage is encoded preferentially by a TTA codon in contrast to the Leu100o of the VRC26.09-like sequences which is encoded by a CTC codon (Fig. 7d). One or more of these convergent mutations are retained in at least one known neutralizing antibody. In addition, the prevalence of replacement mutations at these sites increases over time (Supplementary Fig. 5–6). These convergent CDRH3 signatures may provide further information about the functional importance of these mutations in the protruding CDRH3 loop.

## Discussion

Here, we aimed to aid in the detailed understanding of the maturation pathway of the VRC26 lineage from the CAP256 donor that targets the V1V2 region of the HIV-1 envelope. High resolution information about bNAb evolution trajectories from a UCA is critical for HIV-1 immunogen design. HIV-1 bNAb lineages such as VRC26 are expansive and highly mutated and their reconstructed phylogenies may be difficult to interpret at a refined level. To better address some of these challenges and gain as much insight as possible into the development of the VRC26 lineage, we simultaneously sequenced the exons and introns of lineage members. Obtaining phylogenetic information from both the exon and the intron, the latter which is not under selection pressure, helped us to (i) rapidly establish intraclonal convergence within the CDRH3s of VRC26 antibodies, (ii) identify likely intermediates for the VRC26.19 bNAb having highly divergent CDRH3s and (iii) informatically select intermediate antibodies with critical CDRH3 mutations and that developed heterologous neutralization breadth several months before the earliest bNAbs detected by single B cell cloning.

Using exonic and intronic information together, we were able to differentiate between two important sublineages – the Arg97 + disulfide and the Gly97 + disulfide sublineage – in which the critical Arg97, Cys100a and Cys100q CDRH3 mutations develop and help to stabilize the long CDRH3 loop of VRC26 lineage members. The evolution of these two sublineages had not been explored despite their significance in the lower major bifurcating branch, which contains the broadest VRC26 monoclonals. The informatically-identified GDS-3, GDS-4, and ADS-4 antibodies are the earliest characterized VRC26 intermediates with the disulfide bond and/or Arg97. In addition, these antibodies appear only ~14–25 weeks after the VRC26 lineage first appeared in the CAP256 repertoire. GDS-4, specifically, emerged after only 14 weeks and was able to neutralize 7/14 heterologous strains, providing further evidence that antibodies with appreciable breadth have the potential to be elicited soon after lineage elicitation. This NAb had only ~6.4% nucleotide V gene divergence and had 7 CDRH3 mutations from the UCA other than the Cys100a and Cys100q. The Phe100o CDRH3 mutation is the only one unique to GDS-4 and is present at a site where intraclonal convergence is occurring around the same time, both indicating the possibility of this residue having a particular functional importance. This information sets a new lower boundary of SHM necessary to achieve breadth among lower major bifurcating branch antibodies. The lowest previous SHM level for lower branch bNAbs was ~9% V gene mutation^26^. GDS-(1–4) also constitute a set of antibodies with increasing heterologous breadth and increasing affinity for an early week 34 autologous strain that might be used as scaffolds for stepwise vaccine design. Conversely, this week 34 autologous strain might also serve as an initial complementary Env scaffold, as has been previously suggested^29^.

Identifying late-stage bNAb intermediates is complicated by the likely possibility that CDRH3s may have diverged greatly over long maturation times, often spanning years. Understanding the phylogenetic relationship of lineage members with highly diverse CDRH3s has been a significant challenge^10^. Using both the exon and intron phylogenies we discovered week 119 intermediate antibodies that preceded the development of week 206 VRC26.19 (~46% heterologous neutralization breadth) bNAb-like antibodies^26^ nearly 2 years later. However, the CDRH3 sequences between these week 119 and week 206 antibodies were highly diverse with ~70% amino acid identity. Furthermore, our approach helps confirm the phylogenetic relationship of VRC26.01 sublineage antibodies having diverse CDRH3s, including those with a Tyr insertion. Since insertions and deletions are common in bNAb lineages and may be critical for function^7,10^ understanding when they emerge is of great interest.

Lastly, by using multiple sources of information, particularly the addition of selection-free introns, we identified a number of instances where the same CDRH3 amino acid substitutions appear to have been selected repeatedly along different trajectories, revealing intraclonal convergence. Four of these amino acid mutations were shared with VRC26 bNAbs (VRC26.19 and VRC26.09) that neutralize ~46% of heterologous strains, possibly defining functionally important CDRH3 convergent signatures. In addition, two antibodies with one or more of these mutations (ADS-4 and VRC26.25) have higher affinity to a week 34 autologous viral strain than the other antibodies tested without convergent mutations, suggesting that the identification of convergent mutations might be useful for the fine tuning and optimization of more potent VRC26 bNAbs. The presence of such convergence events during antibody maturation is very challenging to independently confirm or even identify solely by using an exon phylogenetic analysis. The prevalence of intraclonal convergence in other lineages and whether it is selection-driven or a useful developmental mechanism to induce with engineered immunogens will need further evaluation.

## Methods

### Healthy donor B cell isolation

About 50 mL of healthy human donor one (HD1) or healthy donor two (HD2) source leukocytes peripheral blood (Gulf Coast Regional Blood Center, Houston, TX, USA; E5318 product code) was used as a source for separating all nucleated cells from red blood cells using HetaSep (Stemcell Technologies) according to the manufacturer’s protocol. B cells were then extracted and enriched for from the nucleated cells using an EasySep Human CD19 + Positive Selection Kit (Stemcell Technologies) according to the manufacturer’s protocol. The enriched B cells were resuspended in ~500 µL of FACS buffer. These B cells were then stained with a 1:16 dilution of anti-IgD-PE (BD, cat#562024), a 1:16 dilution of anti-CD20-FITC (BD, cat#560962), a ~1:26 dilution of anti-CD19-v450 (BD, cat#560353), a 1:61 dilution of anti-CD3-PerCP-Cy5.5 (Biolegend, cat#300327), and a 1:16 dilution of anti-CD27^−^APC (BD, cat#558664) and then sorted for naive (CD20^+^CD19^+^IgD^+^CD3^−^CD27^−^) and memory (CD20^+^CD19^+^IgD^−^CD3^−^CD27^+^) cells on a FACSAria II.

### CAP256 donor

The CAP256 donor was initially enrolled in the Centre for the AIDS Programme of Research in South Africa (CAPRISA) 002 Acute Infection Study^32^ in KwaZulu-Natal, South Africa in 2004 and samples were drawn between 2005 and 2009. The CAPRISA 002 Acute Infection study complied with all relevant ethical regulations and was approved by the ethics committees of the University of KwaZulu-Natal (E013/04), the University of Cape Town (025/2004) and the University of the Witwatersrand (MM040202). Donor CAP256 provided written consent for the initial study.

### Genomic DNA isolation

Genomic DNA was extracted from CAP256 longitudinal samples of total PBMCs using an AllPrep kit (Qiagen) following the manufacturer’s protocol. The number of cells used for each CAP256 longitudinal time point is listed in Supplementary Table 1. All CAP256 gDNA samples were eluted into 100–200 µL of deionized water. HD1 and HD2 gDNA was isolated using a DNeasy kit (Qiagen) and eluted into 100 µL of elution buffer. Each column was used for a second elution into another 100 µL of elution buffer to collect the remaining gDNA.

### Generation of monoclonal control plasmid template

A human lambda light chain monoclonal control plasmid consisting of a V_λ_ + J_λ_/C amplicon (using IGLV1–51and IGLJ1 germline genes) was amplified from ~150 ng of HD2 gDNA using the primers in Supplementary Table 5. Amplification was done with NEB HF Phusion Polymerase with the outer control generating primers used for the first PCR and the IGLV1–51 leader peptide forward and IGLJ1 300 series reverse primers used for the nested PCR. This amplicon was then cloned and Sanger sequenced.

### High-throughput sequencing

All samples prepared for high-throughput sequencing require a first round PCR of 35 cycles followed by a nested PCR of 25 cycles. The following PCR program is the same for almost all first round and nested PCRs: 98 ^o^C for 5 mins for initial denaturation, 98 ^o^C for 30 s, 55 ^o^C for 30 s, 72 ^o^C for 30 s, and a final extension step of 72 ^o^C for 7 mins and cooled to 4 ^o^C. Annealing temperatures were lowered to 50 ^o^C for the HD1 heavy chain multiplex PCRs. All PCR recipes consist of the following per 25 µL of reaction volume: 5 µL of 5x Phusion Buffer (NEB), 0.5 µL of 10 mM each dNTP mix (NEB), 0.25 µL of HF Phusion Polymerase (NEB) with forward primers, reverse primers, water and template constituting the remaining volume. All first round PCRs utilize forward primers for one or multiple L1 leader peptides while the reverse primer consists of one or more of the 300 series outer intron primers. First round products were consolidated with a DNA cleanup kit (Zymo; with multiple columns if necessary) and diluted 1:10 with 1 µL of this product being used as template per 25 µL of nested PCR reaction volume. All nested PCRs utilize one or multiple FR1 forward primers and one or more of the 250 series inner intron primers. Nested products were cleaned up and consolidated once more and were gel-purified for sizes ~450–600 bp. Each sample received an overall total of 60 cycles before standard Illumina library prep and addition of adapters, followed by Illumina MiSeq 2 × 300 paired-end sequencing.

The lambda monoclonal control sample for high-throughput sequencing was prepared from 80 ng of the generated monoclonal control plasmid template using the FR1 and 250 series primers in Supplementary Table 5. For amplification of the non-monoclonal healthy donor samples (HD1 source gDNA) and VRC26 longitudinal samples, ~200–250 ng of gDNA was used per 25 µL of first round PCR reaction volume. The primers used for the HD1 memory and naive repertoires are listed in Supplementary Table 6. All heavy chain VRC26 first round PCRs used the same L1 leader peptide and 300 series outer intron primer. The first round PCR primers for light chain VRC26 samples are also the same for all time points. The FR1 forward primers used in the nested PCR for each heavy or light chain longitudinal time point were designed to take into account the most prevalent FR1 mutations found in the final datasets of the previously reported RNA-derived sequences^7,26^. Supplementary Data 1 lists the intron and leader peptide primers used for the heavy chain samples along with the FR1 sequences used for the Part I amplification of sequences which utilized only a limited number FR1 nested primers and resulted in the datasets marked as “I” in Supplementary Table 3. A second round of nested PCRs were performed from the first PCR template utilizing the Part II primers listed in Supplementary Data 1 and are marked as the “II” datasets in Supplementary Table 3. A select few of these prepped Part I and Part II datasets were re-sequenced as technical replicates to get a higher read coverage and are marked with an additional “-II” in Supplementary Table 3. All VRC26 longitudinal light chain datasets were only generated and sequenced once with the primers listed in Supplementary Data 1 and with the final sequencing datasets listed in Supplementary Table 4.

The human mBC IGHJ6 intron amplicons were generated by amplifying with a heavy chain FR1 multiplex primer set with a downstream IGHJ6 intron-specific primer in the first PCR. A subsequent nested PCR was performed with an IGHJ6 J gene-specific primer and a nested IGHJ6 intron primer to generate amplicons of IGHJ6 introns that correspond to cells carrying a rearranged IGHJ6-utilizing antibody sequence. The primers that were used are listed in Supplementary Table 7.

### Bioinformatic curation of VRC26 lineage members

Raw MiSeq paired-end reads from the VRC26 heavy chain samples were stitched together using the PEAR (v0.9.10) (http://sco.h-its.org/exelixis/web/software/pear/) paired-read stitching program. FASTX-Toolkit (v36.06) (http://hannonlab.cshl.edu/fastx_toolkit/) was then used to filter for reads that have a quality score of 20 for ≥90% of the read. Sequences were then annotated with IMGT High V-Quest (http://www.imgt.org/HighV-QUEST/)^33^. Reads were retained that aligned to the VRC26 germline V gene (IGHV3–30; as well as IGHV3–30–3 or IGHV3–30–5 to avoid filtering out incorrectly annotated somatic variants) and germline J gene (IGHJ3) ignoring alleles and keeping those that possessed all four framework regions and all three CDRs, and that were productive.

For each longitudinal time point (including Part I and Part II datasets), blastn from BLAST + (version 2.2.28 + ) (https://blast.ncbi.nlm.nih.gov/Blast.cgi) was used to isolate potential VRC26 lineage members by querying for all unique nucleotide CDRH3 sequences from the previously published datasets^7,26^. Sequences that had a nucleotide CDRH3 sequence within 85% identity and 99% query coverage (to allow for soft-clipping) (-max_target_seqs 5000 to ensure full coverage of each database, word size of 28 (default)) to the previous CDRH3 sequences were retained. These sequences were then filtered for only those that contained exact matches for both the forward and reverse primer sites, sorted by abundance (in decreasing order) and then clustered to 96% identity with USEARCH (v8.1.1861) (-sortedby size –maxaccepts 0 –maxrejects 0)^34^. A human lambda light chain monoclonal control (545 bp length amplicon) served to help set the 96% clustering identity cutoff and showed that at least 99.98% of control sample sequences that were able to be aligned with bowtie2 (v2.2.6) (http://bowtie-bio.sourceforge.net/bowtie2/manual.shtml) with an end-to-end alignment were within 96% identity to each other based on their PCR/sequencing error rates (ignoring indels). These alignments were done only after the monoclonal control sequence dataset had undergone the earlier filtering steps to assess the required stringency of clustering only after those previous steps. This is comparable to that used by others^7,10,26^. Only the sequences of the centroids of each cluster output by USEARCH that had a cluster size of more than two members were selected for further processing. These sequences were then aligned using MAFFT (version v7.215) (http://mafft.cbrc.jp/alignment/software/)^35^ (–auto) and manually curated to remove those that had external insertions outside the primer site or that were missing the majority of the intron portion of the read. Only sequences that possessed a CDRH3 matching the Python regular expression C[A-Z]{10,40}WG.G were retained (Supplementary Table 3). The previously inferred UCA sequence^7^ was concatenated to a primer-dictated germline segment of the IGHJ3*02 intron and was inserted into this FASTA file along with all identified and marked triple replicate sequences (see Triple replicate sequence identification). For certain analyses, sequences that were likely PCR chimaeras were also excluded when they could be identified.

The variable region was translated and aligned in amino acids by MAFFT and then reverse translated and re-concatenated to the nucleotide alignment by MAFFT of the intron sequences. The forward and reverse primer sites were then clipped since they are degenerate. One hundred partitioned maximum likelihood trees were then generated using RAxML (version 8.0.26) (http://sco.h-its.org/exelixis/web/software/raxml/)^36^ using the provided GTRCAT approximation. This was done for the variable region alone, the intron alone and by partitioning on both the coding variable region and the non-coding intron region separately. The final trees were optimized under the GAMMA model and the tree with the best likelihood score was used for further analysis. Trees were then visualized with FigTree (http://tree.bio.ed.ac.uk/software/figtree/) and rooted onto the previously inferred UCA, the IGHJ3 intron or the UCA concatenated to the IGHJ3 intron (UCA + J_H3_/C) and ladderized right.

The same procedure was followed for the VRC26 lambda light chain samples except all sequences annotated to the VRC26 germline genes, IGLV1–51 and IGLJ1, were extracted. Sequences with a 92% CDRL3 nucleotide identity to previously published VRC26 light chain sequences^7,26^ were assumed to be members of the lineage since CDRL3s are shorter and less variable. Only one partition tree was generated for the light chain members and the previously inferred light chain UCA^7^ was used to root this tree after being concatenated to the primer-dictated IGLJ1*01 intron sequence segment. Sequences were filtered for those containing a CDRL3 motif C[A-Z]{5,20}F within the last 40 amino acids of the exon (Supplementary Table 4). The same basic filtering and curation steps were also used for all healthy donor samples (Supplementary Table 2). However, the HD1 naive sample did not use the maxrejects = 0 or maxaccepts = 0 clustering termination conditions due to computational intensity.

### Intron allele identification for the VRC26 lineage

The VRC26 lineage germline heavy chain J gene is IGHJ3. There are only two known human IGHJ3 gene exon alleles (IGHJ3*01 and IGHJ3*02). From the IMGT genetic sources, we were able to identify two intronic variants for each of these alleles, which are differentiated by indels or point mutations (Supplementary Data 1). IGHJ3*02 was previously thought to be the J gene allele during the initial recombination event for the VRC26 lineage^7^. All intron sequences of final VRC26 lineage members have a higher identity to IGHJ3*02 Variant 1 than any of the other three variants, except two sequences that, when manually examined, match more appropriately with IGHJ3*02 Variant 1, as expected. Importantly, of the VRC26 lineage sequences, ~50 exact germline IGHJ3*02 Variant 1 allele introns could be found, while none exactly matched any of the other IGHJ3 intronic variants. Likewise, an intron alignment of all IGHJ3 sequences from a CAP256 week 38 dataset (not just from the VRC26 lineage) against IGHJ3*02 Variant 1 showed that all sites mutated ~10% or less except for site 114 at ~50%, indicating that this is only potential site for intronic heterozygosity, with most sequences having either a C or G. All triple replicate sequences and most sublineage sequences have no mutations at this site. Similarly, the VRC26 germline light chain J gene is IGLJ1*01, of which there is only one known allele, and of which we could identify only one full length variant (Variant 1) (Supplementary Table 8). ~30 sequences with introns exactly matching that of the known IGLJ1*01 Variant 1 were found in the final VRC26 lineage light chain sequences. An alignment of all IGLJ1 sequences from a CAP256 week 38 dataset (including sequences from outside the VRC26 lineage) against IGLJ1*01 Variant 1 shows all sites ~30% mutated or lower, with the highest peaks appearing only at hotspots, suggesting intronic homozygosity.

### Triple replicate sequence identification

All sequences identified from each of the sequenced datasets with a ≥98% nucleotide identity and ≥99% query coverage (blastn) to members of the Doria-Rose et al. 2014, 2016 datasets^7,26^ were extracted. Any of these sequences (excluding primer sites) that had duplicate reads in two or more gDNA-derived MiSeq datasets were isolated as those of the highest quality since they have a high identity to members of previously reported RNA-derived datasets and have exact replicates present in gDNA-derived datasets generated through different nested PCRs (Part I and Part II datasets) or re-sequencing (-II datasets). Only gDNA-derived sequences that unambiguously matched with an RNA-derived sequence were considered. If there were multiple matches to an RNA-derived sequence, only those of the highest identity to the RNA-derived sequences were considered. GDS-4 and VRC26.01 fulfill these requirements. While ADS-4 is found with an exact match only within the same dataset, its exon is also found in the previously published RNA-derived raw data from week 59 with only a single nucleotide substitution in the CDR2.

### Selection of heavy and light chains for expression

Sequences from the Gly97 + disulfide and Arg97 + disulfide sublineages with various numbers of shared intronic mutations with either GDS-4 or ADS-4 were chosen for expression to represent the stages of the evolution of each sublineage as best as possible, while minimizing the presence of unshared intronic mutations and investigating various CDRH3 molecular traits. These sequences also exhibited a general phylogenetic relationship in the V_H_ exon tree. Each of these antibodies is labeled as either GDS (Gly97 + disulfide) or ADS (Arg97 + disulfide) with a number.

To estimate complementary light chains for the expression of the selected GDS and ADS heavy chains, we utilized the V_λ_ + J_λ_/C partition ML light chain tree and the V_H_ + J_H_/C partition ML heavy chain tree to incorporate as much genetic information as possible into single trees for pairing phylogenetic branches. Examination of the V_λ_ + J_λ_/C partition light chain tree revealed two major bifurcating branches similar to that of the heavy chain. The lower light chain major bifurcating branch contains sequences with high identity to the light chains of VRC26.01 and VRC26.24 (Supplementary Fig. 4) along with 6/7 triple light chain replicates, all of which are thought to have appeared early in the lineage^7,26^. This suggests that the lower light chain major bifurcating branch is associated with the upper major bifurcating branch of the heavy chain tree. We therefore reasoned that the upper major bifurcating light chain branch corresponded to the lower major bifurcating heavy chain branch (where the chosen heavy chain sequences are located). There are few week 119 and week 206 light chain sequences and many of these appear in the upper major bifurcating branch of the light chain tree, providing further evidence for this reasoning.

We reasoned that the two strongly defined light chain clades of the upper major bifurcating branch might correspond to the strongly defined clades that represent the Arg97 + disulfide and Gly97 + disulfide sublineages. We reasoned that the more extensively mutated Gly97 + disulfide sublineage clade might correspond to the more mutated of these light chain clades, while the less mutated Arg97 + disulfide sublineage clade might correspond to the less mutated of these light chain clades (Supplementary Fig. 4).

For a given heavy chain sublineage, all sequences from the matching light chain clade were binned into intronic divergence levels, and any that had a corresponding divergence to the chosen heavy chain sequence were selected as potential complementary light chain candidates. From these candidates, the sequence sharing the highest number of intronic mutations with the most intronically-mutated sequence furthest along in the light chain clade topology was chosen to help retain the phylogeny of the light chain clade as closely as possible. If there were multiple candidates from this pool that met these two criteria, one was chosen at random (using the random module implemented in Python 2.7.6).

### Antibody expression

Sequences for heavy and light chains were codon optimized for mammalian expression, synthesized (GenScript), and cloned in the pVRC8400 vector. Equal amounts of heavy and light chain plasmid were co-transfected with Expi293F cells (Thermo Fisher) according to the cell manufacturer’s directions. Following a 6-day incubation, cells were pelleted by centrifugation and supernatant was filtered through 0.22 μm Stericup filter units (EMD Millipore) and then applied to a column containing a 2 mL bed of Protein A Sepharose Fast Flow (GE Healthcare, Chicago, IL) equilibrated with PBS. The column was washed with Protein A IgG Binding Buffer, and antibody was eluted with Pierce IgG Elution Buffer (Thermo Scientific) and pH neutralized with 1 M Tris pH 8 solution. Antibodies were buffer-exchanged to PBS overnight using 10,000 MWCO dialysis cassettes (Thermo Fisher).

### Silent and replacement mutation plots

All VRC26 heavy chain lineage sequences were aligned as amino acids and this alignment was then reverse translated into a nucleotide alignment. Sequences were split into their respective time points and all sequences were compared to the UCA (including the CDRH3) to determine silent and replacement mutations. Indels were ignored for this analysis. Their frequency was plotted as the percent of sequences in each time point containing this mutation.

### Generating divergence plots

To generate the healthy donor divergence plots, only curated sequences were used. The introns from these sequences were aligned with MAFFT to each of the relevant germline introns, with the lowest divergence value generated from each of these alignments taken to be the final value. Intronic divergence was calculated as the number of SHM events (number of point mutations + number of indel events) divided by the length of the germline intron portion of a theoretical amplicon (excluding the primer site). V gene divergence values were calculated using IMGT annotated V gene mutations. The most prevalent alleles by exact match read counts in the naive dataset were used. The same procedure was used for the VRC26 lineage longitudinal divergence plots, except these plots are generated from all datasets corresponding to a single time point alone and then curating with pooling across time points. All of these sequences were aligned only to the IGHJ3*02 Variant 1 intron or to the IGLJ1*01 Variant 1 intron, which were chosen as described in Intron allele identification for the VRC26 lineage.

### IGHJ6 intron mutation distribution plot

The IGHJ6 intron mutation distribution frequency plot was generated using the same filtering stringency as other samples except without IMGT filtering steps. Sequences were clustered using USEARCH to a 96% identity (centroids only taken from clusters with more than two members) and were required to have exact primer matching. Sequences were aligned using MAFFT (–auto) to the germline IGHJ6*03 Extended Variant 1 allele sequence to determine the mutation frequency at various sites. This allelic variant was chosen since HD1 alignments indicate the donor is likely heterozygous for IGHJ6*03 Extended Variant 1 and IGHJ6*02 Extended Variant 2 (Supplementary Data 1) with a single nucleotide polymorphism at site 121.

### Neutralization assays

Env pseudoviruses were generated through the cotransfection of a pSG3ΔEnv backbone plasmid and a plasmid encoding a full Env gp160 in a 3:1 ratio in 293 T cells using the FuGENE transfection reagent (Promega). Media was replaced the day following transfection and cells were allowed to incubate overnight once more. The next day the media was then filtered with a 0.45 µm Steriflip unit (EMD Millipore) to collect pseudovirus. 40 µL of diluted pseudovirus was mixed with 10 µL of serially-diluted antibody and incubated for 30 min at 37 ^°^C. 20 µL of TZM-bl cells at a concentration of 10,000 cells/well were then added and incubated overnight at 37^o^C with or without DEAE-Dextran. 100 µL of fresh DMEM was added and the samples which were once again incubated overnight. 50 µL of Steadylite Plus Reporter Gene Assay System (PerkinElmer) was then added, plates were shaken for 15 min and luminescence was measured. Neutralization data is reported as IC_50_ values (µg/mL) and were calculated from measuring the reduction in luminescence by the addition of various dilutions of each antibody. Percent inhibition was defined as 100 times the difference in average RLU between cell only controls and samples with antibodies added divided by the difference in average RLU between virus controls and cell only controls.

The pseudoviruses used for these assays included heterologous strains from Clade A (BB201.B42, BG505.W6M.C2, KER2008.12, KER2018.11, Q23.17), Clade AE (CM244.ec1), Clade AG (T250–4), Clade B (WITO.33), Clade BC (CH070.1), Clade C (CAP210.E8, ZM197.7, ZM233.6, ZM53.12), and Clade D (3016.v5.c45)^7,26^. Pseudoviruses also included autologous strains from week 34 (CAP256.3.11.80, CAP256.3.11.81, CAP256.3.11.77, CAP256.3.11.18, and CAP256.3.11.22), week 38 (CAP256.3.12.38), week 48 (CAP256.3.14.8, CAP256.3.14.17, CAP256.3.14.18), week 59 (CAP256.3.16.2, CAP256.3.16.4, and CAP256.3.16.10b), week 94 (CAP256.4.19.A3, CAP256.4.19.F4) or the superinfecting strain (CAP256.206.C9)^29^.

### Surface plasmon resonance

Binding of CAP256.3.11.80 SOSIP Env trimer to VRC26 lineage members (UCA, GDS-(1–4), ADS-4 and VRC26.25) was measured by SPR with single-cycle kinetics. Antibodies were captured on an anti-Fc surface and Env trimer was flowed at 50 ml/min in HBS-EP buffer. Concentrations of Env trimer ranging from 1 nM to 500 nM were measured in a single cycle dilution series and adjusted within the affinity limits of each lineage member.

### Statistical analysis

Two-tailed Mann–Whitney *U* statistical tests (assuming non-normality, independent groups and observations, ordinal or continuous dependent variable) were performed using the implemented Mann–Whitney *U* test function made available through the scipy.stats module of Python 2.7.6.

### Code availablity

Custom scripts have been deposited in GitHub.

## Electronic supplementary material

Supplementary Information

Description of Additional Supplementary Files

Supplementary Data 1

## Data Availability

Raw sequencing datasets for all samples have been deposited in the NCBI Short Read Archive under accession code SRP124539. Synthesized monoclonal antibody sequences and intermediate sequences have been deposited in Genbank under accession codes MH409941-MH409952. VRC26 heavy and light chain lineage sequences have been deposited in Genbank under accession codes MH407745-MH409383 and MH409384-MH409940. Raw FACS data has been deposited in FlowRepository under ID FR-FCM-ZYPZ.
