# Supplementary material for: Sequencing HIV-neutralizing antibody exons and introns reveals detailed aspects of lineage maturation
Source: Nat Commun. 2018 Oct 8;9:4136. doi: 10.1038/s41467-018-06424-6 (PMC6175870; doi:10.1038/s41467-018-06424-6)
Supplement: Supplementary file 1 — Supplementary Information [file 41467_2018_6424_MOESM1_ESM.docx]

**Supplementary Information**

**Sequencing HIV-neutralizing antibody exons and introns reveals detailed aspects of lineage maturation**

**Johnson et al.**

**Supplementary Figure 1**

**
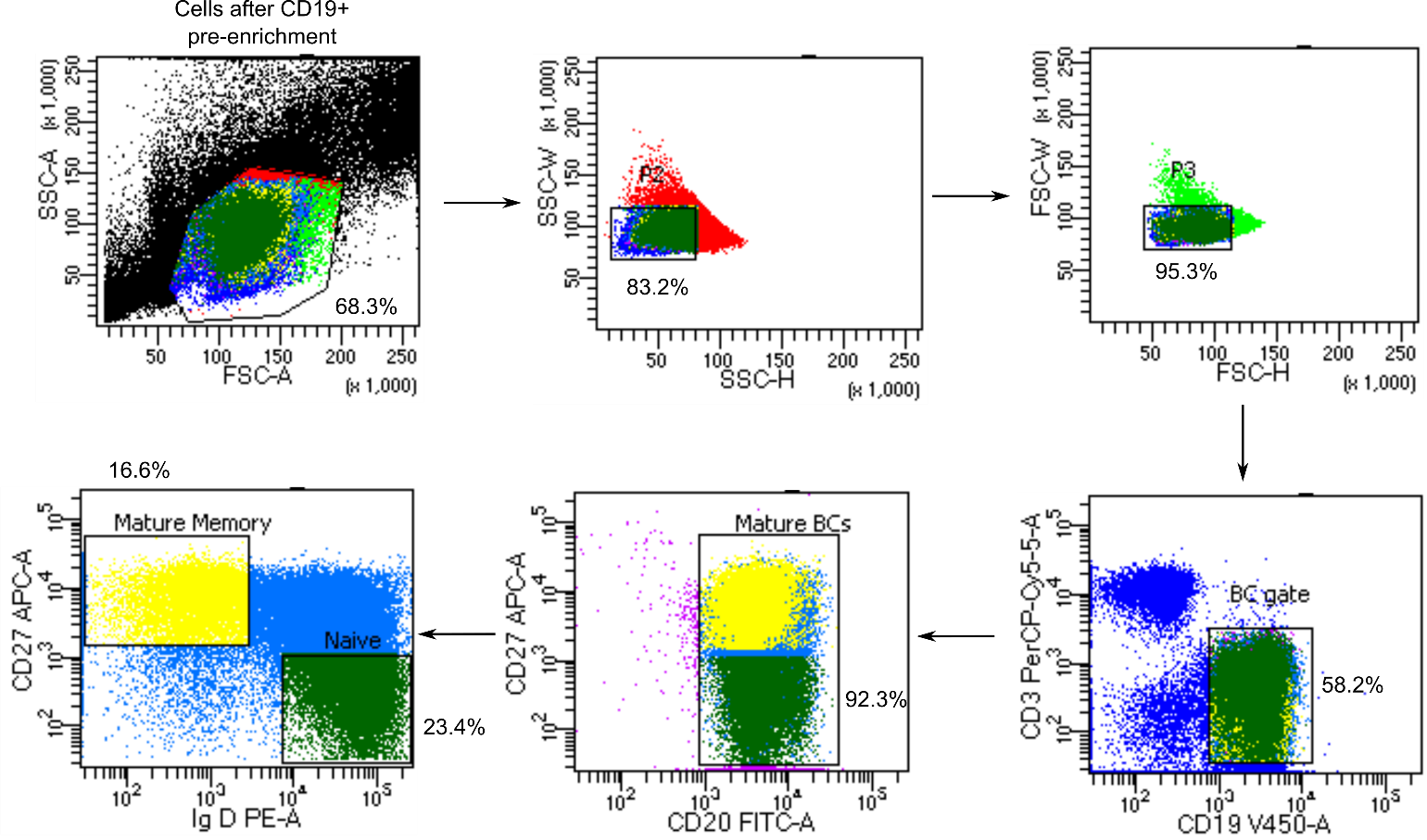
**

**Supplementary Figure 1. Gating strategy for isolating healthy donor B cells.** Gates are shown in sequential order with the percent of the parent population shown. Naive (CD20^+^CD19^+^IgD^+^CD3^-^CD27^-^; gate labeled as Naive) and memory B cells (CD20^+^CD19^+^IgD^-^CD3^-^CD27^+^; gate labeled as Mature Memory) were isolated from cells that went through a CD19+ pre-enrichment step. These two final populations were used to generate the datasets for the plots in **Fig. 1c, d**. P2, P3, BC gate and Mature BCs refer to earlier gates in the strategy.

**Supplementary Figure 2**

**
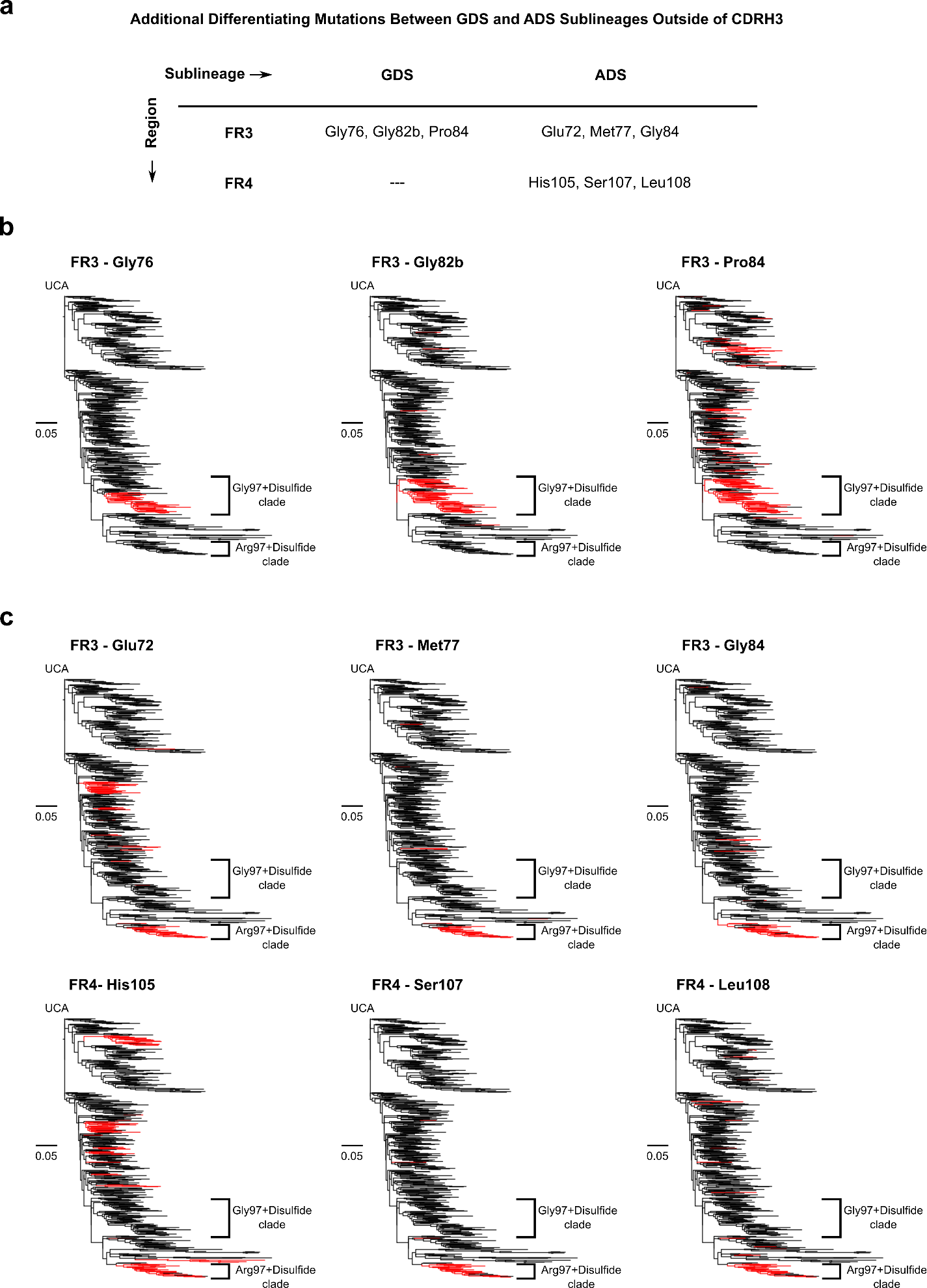
**

**Supplementary Figure 2. Mutations in GDS and ADS sublineages outside of the CDRH3.** (**a**) Mutations outside of the CDRH3 that are prevalent within either the GDS or ADS sublineage. (**b**) V_H_ exon ML tree highlighted for the presence of mutations that are generally present in the GDS sublineage but not in the ADS sublineage. (**c**) V_H_ exon ML tree highlighted for the presence of mutations that are generally present in the ADS sublineage but not in the GDS sublineage. Mutations are numbered according to Kabat numbering. (GDS=Gly97+Disulfide and ADS=Arg97+Disulfide)

**Supplementary Figure 3**

**
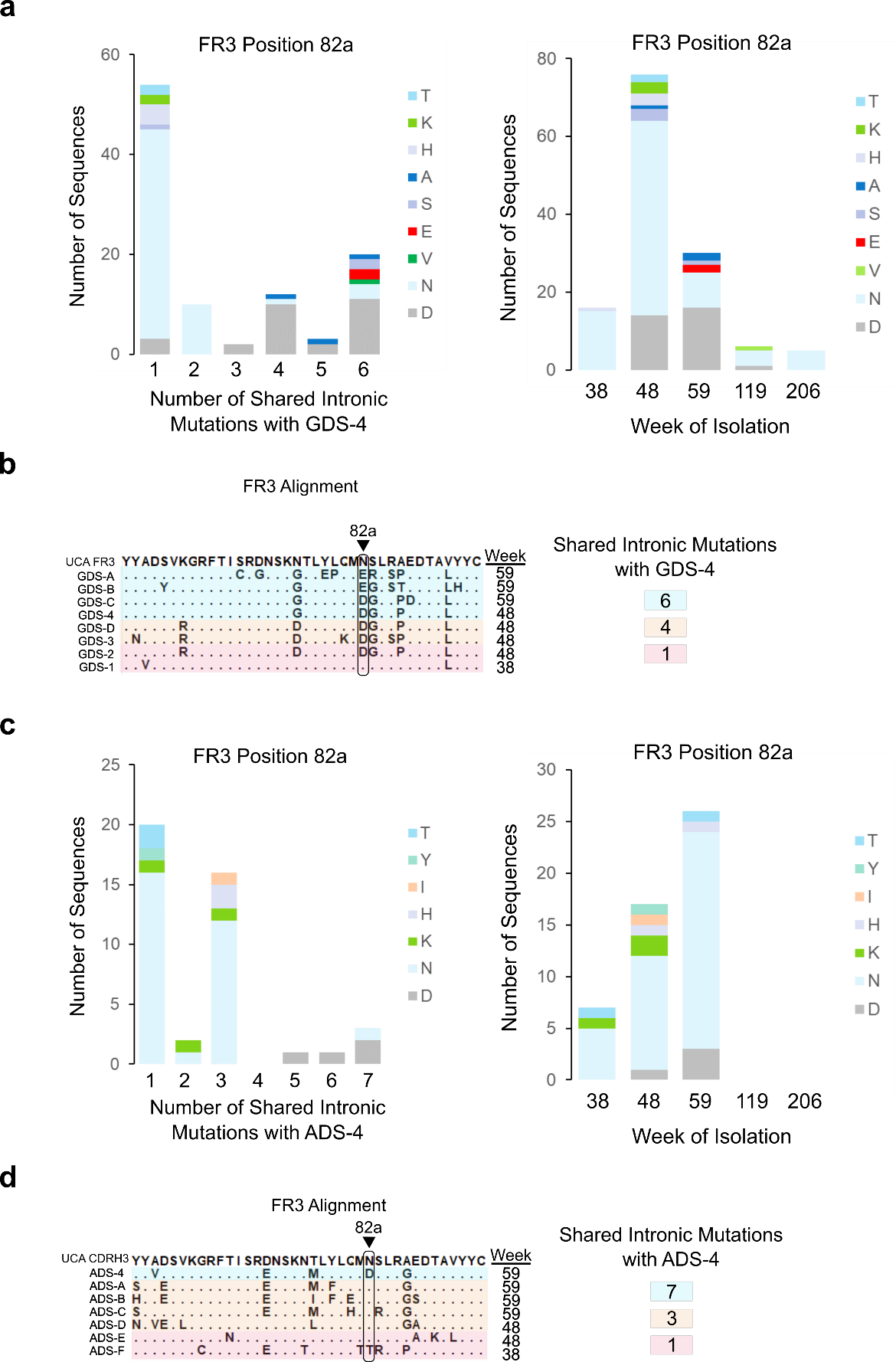
**

**Supplementary Figure 3. An additional site for mutational convergence in FR3.** (**a**) An Asp82a mutation becomes more prevalent within the GDS sublineage over time and as the number of intronic mutations shared with GDS-4 increases. (**b**) FR3 alignment of the GDS sublineage representative sequences shown in **Fig. 3**. (**c**) An Asp82a mutation also becomes more prevalent within the ADS sublineage over time and as the number of intronic mutations shared with ADS-4 increases. (**d**) FR3 alignment of the ADS sublineage representative sequences shown in **Fig. 4**. (GDS=Gly97+Disulfide and ADS=Arg97+Disulfide)

**Supplementary Figure 4**


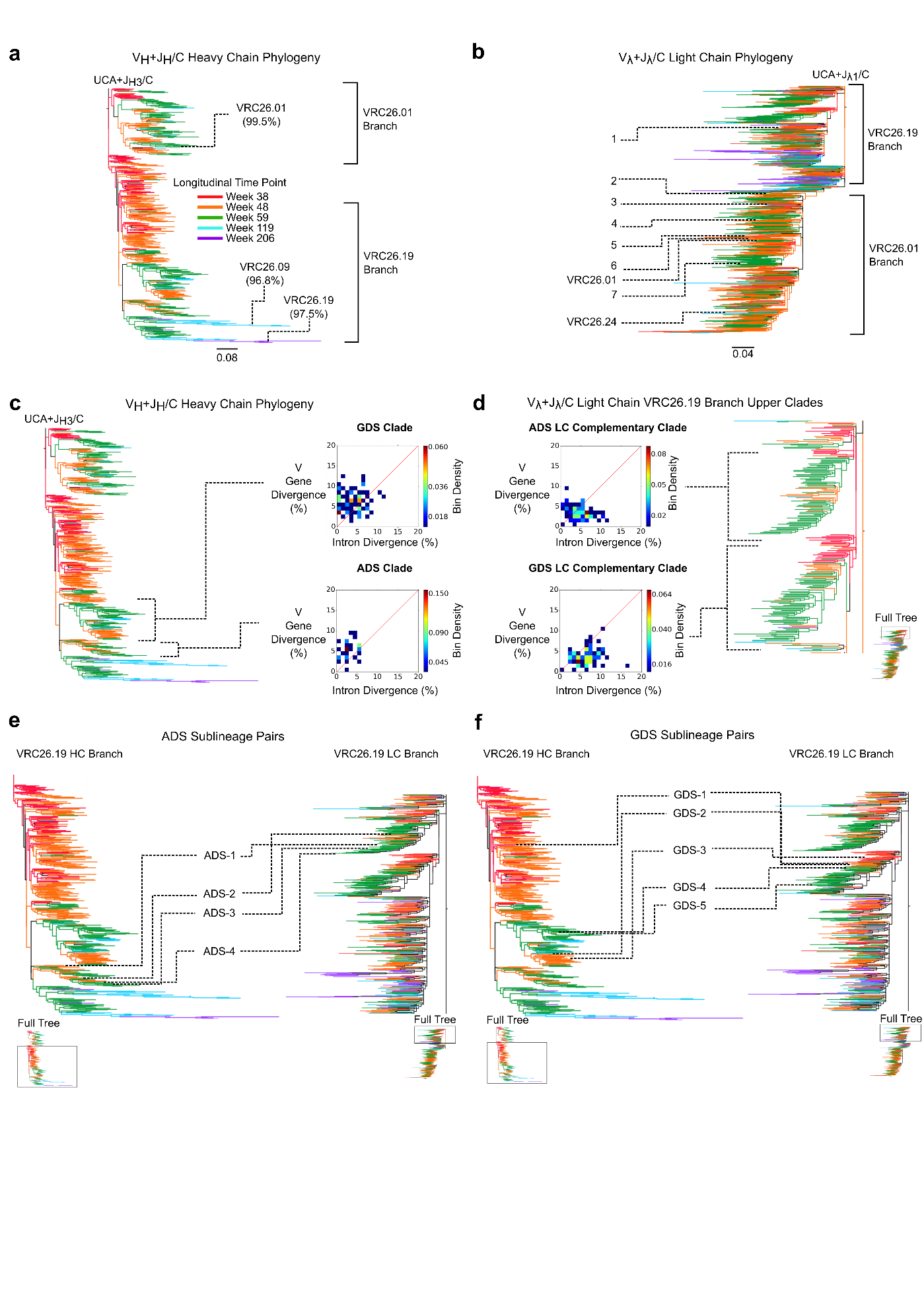


**Supplementary Figure 4. Selection of complementary light chain sequences.** (**a**) The V_H_+J_H_/C partition ML tree with sequences with high identity to previously characterized bNAbs (exon nucleotide percent identity shown). The two major branches of the bifurcating tree are labeled as either VRC26.01 branch or VRC26.19 branch. (**b**) The V_λ_+J_λ_/C partition ML tree with light chain triple replicate sequences labeled as numbers. The two major branches of the tree are labeled as VRC26.01 branch or VRC26.19 branch as before, but with the lower branch containing VRC26.01-like and VRC26.24-like sequences. The orientation of the branches is possibly due to under-sampling of later time points. (**c**) The V_H_+J_H_/C partition ML tree with the major clades of the ADS sublineage and the GDS sublineage labeled. The intronic divergence of the GDS sublineage clade is shown to be more extensive than that of the ADS sublineage clade. (**d**) The two most defined clades of the light chain VRC26.19 branch. The lower of these clades has a more extensive intronic divergence and thus was assumed to be likely complementary with the more extensively mutated GDS sublineage (GDS LC Complementary Clade). The other clade was assumed to be complementary to the ADS sublineage (ADS LC Complementary Clade). (**e**) The VRC26.19 branches of both the heavy and light chain trees are shown. Heavy chain sequences were matched with light chains based on intron mutation load within the assumed complementary clade while also trying to retain light chain intronic phylogeny as closely as possible. (**f**) Same as in (**e**) except for the GDS sublineage. Only ADS-4 and GDS-(1-4) expressed well enough for testing. (GDS=Gly97+Disulfide and ADS=Arg97+Disulfide)

**Supplementary Figure 5**

**
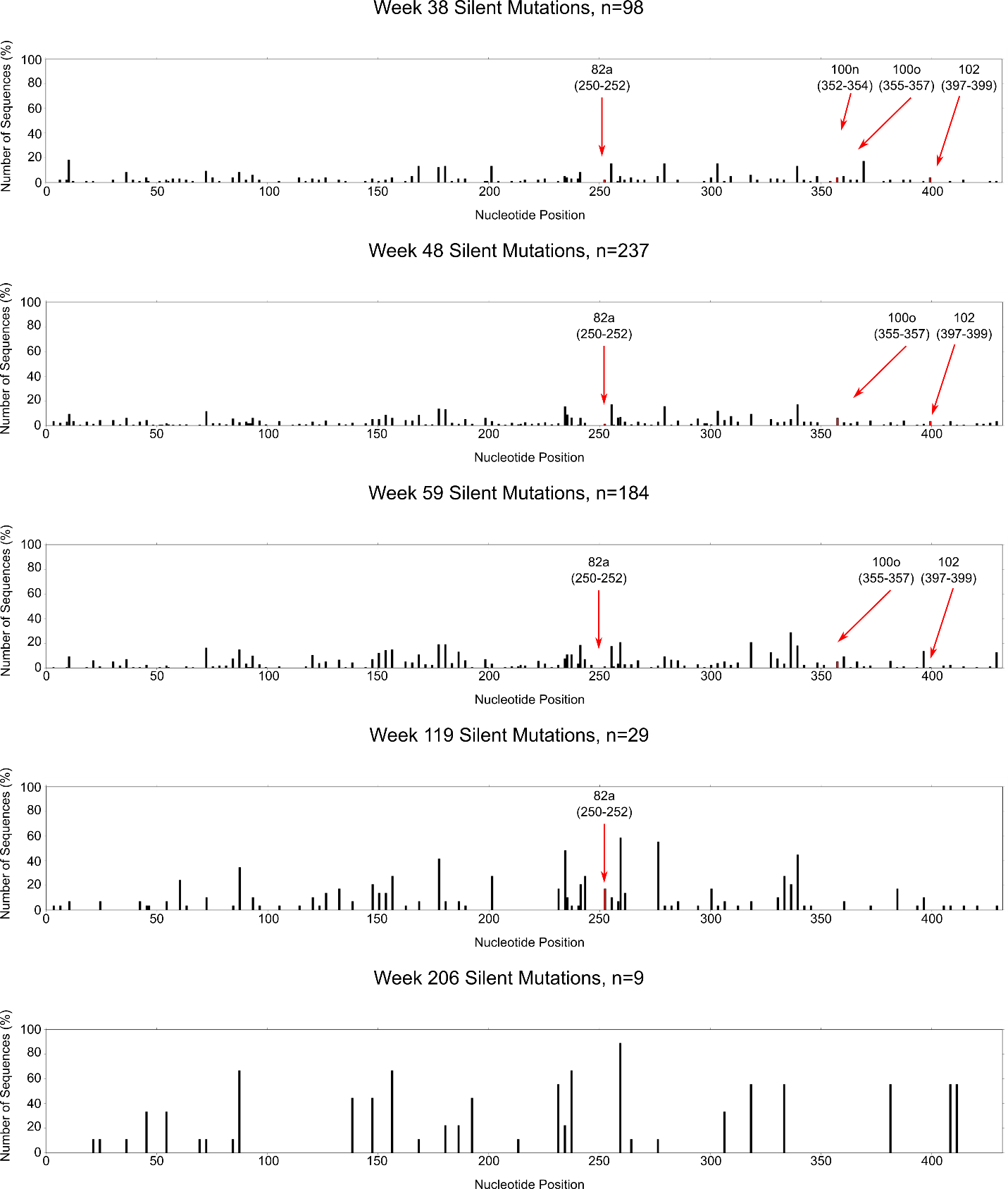
**

**Supplementary Figure 5. VRC26 lineage heavy chain silent mutations across time points.** All mutations, including those in the CDRH3 are compared to the UCA. Nucleotide sites that encode for convergent mutations are highlighted in red and labeled with their Kabat amino acid position and nucleotide positions in parentheses. Number of sequences = n. Week 38: n=98, Week 48: n=237, Week 59: n=184, Week 119: n=29, Week 206: n=9.

**Supplementary Figure 6**

**
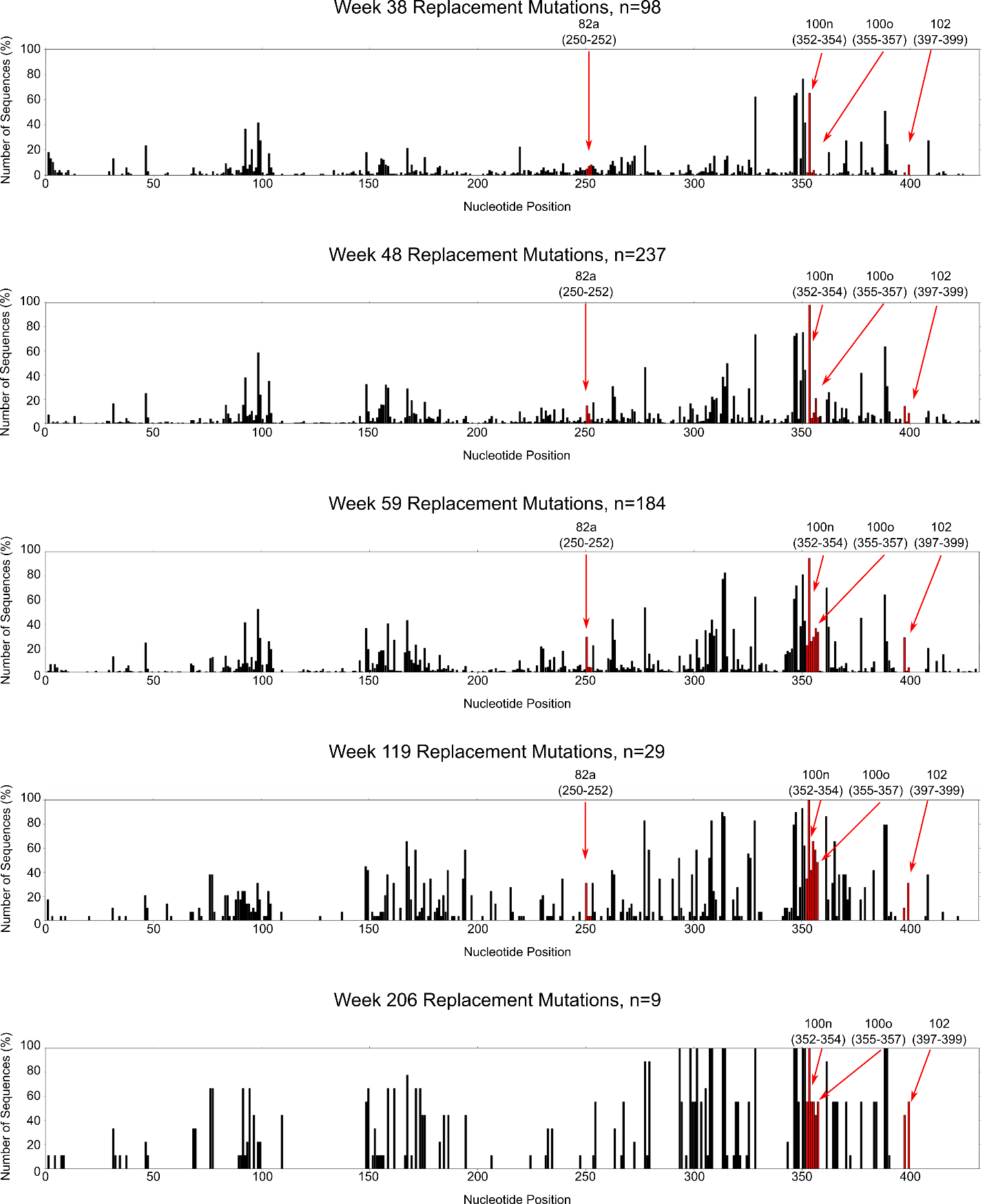
**

**Supplementary Figure 6. VRC26 lineage heavy chain replacement mutations across time points.** All mutations, including those in the CDRH3 are compared to the UCA. Nucleotide sites that encode for convergent mutations are highlighted in red and labeled with their Kabat amino acid position and nucleotide position in parentheses. Number of sequences = n. Week 38: n=98, Week 48: n=237, Week 59: n=184, Week 119: n=29, Week 206: n=9.

| Sample | | Total processed gDNA (µg) | Estimated number of cells processed (sorted, counted or estimated from µg of gDNA) |
| --- | --- | --- | --- |
| VRC26 Week 38 Heavy Chain | 26 | | 7,200,000 PBMCs |
| VRC26 Week 48 Heavy Chain | 35 | | 8,350,000 PBMCs |
| VRC26 Week 59 Heavy Chain | 16 | | 7,500,000 PBMCs |
| VRC26 Week 119 Heavy Chain | 6.9 | | 5,000,000 PBMCs |
| VRC26 Week 206 heavy Chain | 12 | | 6,500,000 PBMCs |
| VRC26 Week 38 Light Chain | 10.4 | | 2,880,000 PBMCs |
| VRC26 Week 48 Light Chain | 24.5 | | 5,845,000 PBMCs |
| VRC26 Week 59 Light Chain | 10 | | 4,687,000 PBMCs |
| VRC26 Week 119 Light Chain | 4.83 | | 3,500,000 PBMCs |
| VRC26 Week 206 Light Chain | 8 | | 4,333,000 PBMCs |
| Healthy Donor mBC Heavy | 0.678 | | 219,000 mBCs |
| Healthy Donor Naïve B Cells Heavy | 0.802 | | 259,000 Naïve B Cells |
| Healthy Donor mBC IGHJ6 Intron | 0.31 | | 100,000 mBCs |

**Supplementary Table 1.** Number of cells processed in all samples. PBMCs = peripheral blood mononuclear cells.

| Curation step | HD1 HC memory | HD1 HC naive | HD1 IGHJ6 |
| --- | --- | --- | --- |
| Raw reads | 1,706,960 | 2,141,597 | 1,186,830 |
| PEAR stitch, FASTX q20p90 | 1,036,657 | 1,351,641 | 317,555 |
| IMGT full, productive or reoriented (e.g. IGHJ6) | 599,488 | 905,641 | 169,704 |
| Primer matching, N removal | 346,583 | 537,509 | 88,998 |
| 96% USEARCH clustering | 89,679 | 291,491 | 34,790 |
| Centroids of cluster size >=2 | 19,704 | 47,372 | 4,796 |

**Supplementary Table 2.** Healthy donor sample bioinformatic curation steps and filtering with the number of sequences retained at each step. HD1 refers to healthy donor 1. IGHJ6 refers to the IGHJ6 intron dataset from HD1.

| Curation step | Week 38 I | Week 48 I | Week  59 I | Week 119 I | Week 206 I | Week 38 II | Week 48 II | Week  59 II | Week 119 II | Week 206 II | Week 206  I-II | Week 206  II-II | Week 119  I-II | Week 119  II-II | Total across time points |
| --- | --- | --- | --- | --- | --- | --- | --- | --- | --- | --- | --- | --- | --- | --- | --- |
| Raw reads | 673,315 | 655,757 | 1,163,364 | 815,171 | 1,378,357 | 844,749 | 781,738 | 1,175,169 | 842,538 | 1,111,139 | 1,321,631 | 944,965 | 899,374 | 950,114 | 13,557,381 |
| PEAR stitch, FASTX q20p90 | 596,920 | 597,725 | 1,026,273 | 740,689 | 1,235,888 | 770,546 | 715,570 | 1,072,527 | 771,164 | 1,021,940 | 1,156,452 | 805,031 | 828,535 | 885,556 | 12,224,816 |
| IMGT IGHV3-30/IGHJ3, full, productive | 108,622 | 144,414 | 170,277 | 194,794 | 220,544 | 186,739 | 183,335 | 242,857 | 204,528 | 259,956 | 205,502 | 209,866 | 226,066 | 242,073 | 2,799,573 |
| BLASTN lineage (85% CDRH3, 99% qcovs, word size 28) | 444 | 1,669 | 1,176 | 83 | 34 | 956 | 1,667 | 2,512 | 96 | 16 | 35 | 15 | 138 | 176 | 9,017 |
| Primer matching | - | - | - | - | - | - | - | - | - | - | - | - | - | - | 7,462 |
| 96% USEARCH clustering | - | - | - | - | - | - | - | - | - | - | - | - | - | - | 1,825 |
| Centroids of cluster size >=2 | - | - | - | - | - | - | - | - | - | - | - | - | - | - | 579 |
| Manual curation | - | - | - | - | - | - | - | - | - | - | - | - | - | - | 579 |
| Final curated with CDRH3 motif match | - | - | - | - | - | - | - | - | - | - | - | - | - | - | 558 |

**Supplementary Table 3.** VRC26 heavy chain bioinformatic curation steps and filtering.

| Curation step | Week 38 LC | Week 48 LC | Week 59 LC | Week 119 LC | Week 206 LC | Total across time points |
| --- | --- | --- | --- | --- | --- | --- |
| Raw reads | 916,046 | 990,855 | 956,680 | 846,332 | 960,002 | 4,669,915 |
| PEAR stitch , FASTX q20p90 | 819,264 | 865,349 | 834,725 | 731,773 | 837,872 | 4,088,983 |
| IMGT IGLV1-51/IGLJ1, full, productive | 444,566 | 507,988 | 656,401 | 496,662 | 636,833 | 2,742,450 |
| BLASTN lineage (92% CDRL3, 99% qcovs, word size 28) | 15,370 | 38,112 | 19,961 | 3,570 | 3,244 | 80,257 |
| Primer matching | - | - | - | - | - | 70,163 |
| 96% USEARCH clustering | - | - | - | - | - | 3,674 |
| Centroids of cluster size >=2 | - | - | - | - | - | 1,933 |
| Manual curation | - | - | - | - | - | 1,907 |
| Final curated with CDRL3 motif match | - | - | - | - | - | 1,640 |

**Supplementary Table 4.** VRC26 light chain (LC) curation steps and filtering.

| HUMAN LAMBDA MONOCLONAL CONTROL GENERATING PRIMERS | | |
| --- | --- | --- |
| Human Lambda Outer Control Generating Primers | | **Final Concentration (nM)** |
| HVL1-51-C-GEN-FWD | GGAGGGTCTGATTTGCATGGATGGAC | 400 |
| HIGKJ1-C-GEN-REV | CTCCCTTTCTGACAATTGACCAAGGC | 400 |
| Human Lambda L1 Peptide Forward | |  |
| HVL-VL1-51-L1-FWD | ATGACCTGCTCCCCTCTCCT | 400 |
| Human Lambda FR1 Forward |  |  |
| hVL1-51-fwd | CAGTCTGTGTTGACGCAGCCG | 1000 |
| Human Lambda Intron 300 Series Reverse | |  |
| HIGLJ1-INTRON-300-REV | CCTGCCCAATCCCAGCTCA | 400 |
| Human Lambda Intron 250 Series Reverse | |  |
| HIGLJ1-INTRON-250-LA | GGACCAGGCTGCATCCGG | 1000 |

**Supplementary Table 5.** Human lambda monoclonal control primers.

| HUMAN MULTIPLEX INTRONIC HEAVY CHAIN REPERTOIRE PRIMERS | | |
| --- | --- | --- |
| L1 Peptide Forward |  | **Final Concentration (nM)** |
| HVH1/7-L1-A | CCATGGACTGSACCTGGA | 62.4 |
| HVH1/7-L1-B | ATGGACTGGATTTGGAGGAT | 62.4 |
| HVH1/7-L1-C | GGTTCCTCTTTGTGGTGGC | 62.4 |
| HVH2-L1-A | ATGGACAYACTTTGYTCCAC | 62.4 |
| HVH2-L1-B | ATGGACACACTTTGCTACAC | 62.4 |
| HVH3-L1-A | CCATGGAGTTTGGGCTGAGC | 62.4 |
| HVH3-L1-B | GCTGGGTTTTCCTTGTTGCT | 62.4 |
| HVH3-L1-C | GCTGGCTTTTTCTTGTGGCT | 62.4 |
| HVH3-L1-D | AGT GTG AGT GGA CRT GAG TG | 62.4 |
| HVH3-L1-E | CCATGGAGTTGGGACTGAGC | 62.4 |
| HVH3-L1-F | GAT TGC TGA GCT GTT CTG TGC | 62.4 |
| HVH3-L1-G | GGT GTA CTA GAG ATA CTG AGT GTG A | 62.4 |
| HVH4-L1-A | ATGAARCACCTGTGGTTCTT | 62.4 |
| HVH4-L1-B | ATGAAACATCTGTGGTTCTTCC | 62.4 |
| HVH5-L1 | ATGGGGTCAACCGCCATCCT | 62.4 |
| HVH6-L1 | ATGTCTGTCTCCTTCCTCAT | 62.4 |
| FR1 Forward |  |  |
| HVH1-FWD | CAGGTCCAGCTKGTRCAGTCTGG | 50 |
| HVH157-FWD | CAGGTGCAGCTGGTGSARTCTGG | 50 |
| HVH2-FWD | CAGRTCACCTTGAAGGAGTCTG | 50 |
| HVH3-FWD | GAGGTGCAGCTGKTGGAGWCY | 50 |
| HVH4-FWD | CAGGTGCAGCTGCAGGAGTCSG | 50 |
| HVH4-DP63-FWD | CAGGTGCAGCTACAGCAGTGGG | 50 |
| HVH6-FWD | CAGGTACAGCTGCAGCAGTCA | 50 |
| HVH3N-FWD | TCAACACAACGGTTCCCAGTTA | 50 |
| Intron 300 Series Reverse |  |  |
| HIGHJ1-INTRON-300-A | TGCCCTCCTGCTTCTCCCATACAA | 142.8 |
| HIGHJ2-INTRON-300-A | TGTGGAGGGTCCCTGACGGG | 142.8 |
| HIGHJ3-INTRON-300-A | CCCAAGGTCATTTTGTCCCCAGCA | 142.8 |
| HIGHJ4-INTRON-300-A | GCCACAGAGAGACCCGTGCT | 142.8 |
| HIGHJ4-INTRON-300-B | GACACGAACCTCCGCCTAGGG | 142.8 |
| HIGHJ5-INTRON-300-A | GTGAAGCGGAGAGAGGTCACC | 142.8 |
| HIGHJ6-INTRON-300-A | CAGGCTCAGTTACTCCATCAGACGC | 142.8 |
| Intron 250 Series Reverse |  |  |
| HIGHJ1-INTRON-250-A | AAAAACACACCCTCCGCCCT | 50 |
| HIGHJ2-INTRON-250-A | TCTGGCATGCAGCCCATGGC | 50 |
| HIGHJ3-INTRON-250-A | CCCAAACAGCCGGAGAAGGAG | 50 |
| HIGHJ4-INTRON-250-A | GCATCCTCCTGAGCCCCC | 50 |
| HIGHJ4-INTRON-250-B | TGTTGCCTCAGGCCTCCTG | 50 |
| HIGHJ5-INTRON-250-A | GGGTTCCCATTCGAAGGGTCC | 50 |
| HIGHJ6-INTRON-250-A | CCCAGTGCCCATCCCCTC | 50 |
| HIGHJ6-INTRON-250-B | CCGTCCCCTCTGGCCAGT | 50 |

**Supplementary Table 6.** Human heavy chain multiplex repertoire primers.

| HUMAN HEAVY CHAIN IGHJ6 REARRANGED IGHJ6 INTRON MUTATION DISTRIBUTION | | |
| --- | --- | --- |
| FR1 Forward |  | **Final Concentration (nM)** |
| HVL1-FWD-OE | CAGTCTGTSBTGACGCAGCCGCC | 125 |
| HVL1459-FWD-OE | CAGCCTGTGCTGACTCARYC | 125 |
| HVL15910-FWD-OE | CAGCCWGKGCTGACTCAGCCMCC | 125 |
| HVL2-FWD-OE | CAGTCTGYYCTGAYTCAGCCT | 125 |
| HVL3-FWD-OE | TCCTATGWGCTGACWCAGCCAA | 125 |
| HVL-DPL16-FWD-OE | TCCTCTGAGCTGASTCAGGASCC | 125 |
| HVL3-38-FWD-OE | TCCTATGAGCTGAYRCAGCYACC | 125 |
| HVL6-FWD-OE | AATTTTATGCTGACTCAGCCCC | 125 |
| HVL78-FWD-OE | CAGDCTGTGGTGACYCAGGAGCC | 125 |
| IGHJ6 J Gene Primer Forward |  |  |
| JH6-02-DIST | CCACGGTCACCGTCTCCTCA | 400 |
| IGHJ6 Intron Outer Reverse |  |  |
| JH6-02-Intron-DIST-1 | TCAATTCCAGACACATATCACTCATGGG | 1000 |
| IGHJ6 Intron Inner Reverse |  |  |
| JH6-DIST-2-All-Alleles | ACTGAGGTCCTGGAGCCTCC | 400 |

**Supplementary Table 7.** IGHJ6 intron mutation distribution primers. See separate excel file.

| **LAMBDA LIGHT CHAIN INTRON ALLELIC VARIANTS** |
| --- |
| **>IGLJ1*01 Variant 1 [D87023.1; X51755.1; AC245028.2]** |
| GTAAGTGGCTCTCAACCTTTCCCAGCCTGTCTCACCCTCTGCTGTCCCTGGAAAATCTGTTTTCTCTCTCTGGGGCTTCCTCCCCTCTGTCCTCCCAGCCTTAAGCACTGACCCTTACCTTTCTCCATGGGGCCTGGAGGAGGTGCATTAGTCTCCGGGTAACCGGCAGGAAGGGCCTCCACAGTGGGAGCAGCCGGATGCAGCCTGGTCC |
| **>IGLJ1*01 Partial Variant 2 [X06877.1]** |
| GTAAGTGGCTCTCAACCTTTCCCAGCCTGTCTCACCCTCTGCTGTCCCTAGAAAATCTGTTTTCTGTCTCTGGGGCTTCCTCCTCTGTCCTCCCAGCCTTAAGCACTGACCCTTACCTTTCTCCATGGGGCCTGGAGGAGGTGCATTAGCCCCGGGTAACCGGCAGGAAGGGCCTCCACAGTGGGAGCAGCCTG |

**Supplementary Table 8.** Human lambda light chain intron allelic variants. Various genetic source accession numbers are listed for each.
