# Supplementary material for: Sequencing HIV-neutralizing antibody exons and introns reveals detailed aspects of lineage maturation
Source: Nat Commun. 2018 Oct 8;9:4136. doi: 10.1038/s41467-018-06424-6 (PMC6175870; doi:10.1038/s41467-018-06424-6)
Supplement: Supplementary file 2 — Description of Additional Supplementary Files [file 41467_2018_6424_MOESM2_ESM.docx]

Supplementary Data File Legends.

**Spreadsheet 1.** VRC26 heavy chain Part I primers.

**Spreadsheet 2.** VRC26 heavy chain Part II primers.

**Spreadsheet 3.** VRC26 light chain primers.

**Spreadsheet 4.** Human heavy chain intron allelic variants. Various genetic source accession

numbers are listed for each.
